# ERK5 Inhibition Induces Autophagy-Mediated Cancer Cell Death by Activating ER Stress

**DOI:** 10.3389/fcell.2021.742049

**Published:** 2021-11-04

**Authors:** Andrés Gámez-García, Idoia Bolinaga-Ayala, Guillermo Yoldi, Sergio Espinosa-Gil, Nora Diéguez-Martínez, Elisabet Megías-Roda, Pau Muñoz-Guardiola, Jose M. Lizcano

**Affiliations:** ^1^ Departament de Bioquímica i Biologia Molecular and Institut de Neurociències, Universitat Autònoma de Barcelona (UAB), Barcelona, Spain; ^2^ Protein Kinases in Cancer Research, Vall Hebron Institut de Recerca (VHIR), Barcelona, Spain

**Keywords:** autopaghy, ERK5 kinase, UPR – unfolded protein response, cancer cell survival, endoplamic reticulum stress, apoptosis, antitumor drug, MAPK signal pathway

## Abstract

Autophagy is a highly conserved intracellular process that preserves cellular homeostasis by mediating the lysosomal degradation of virtually any component of the cytoplasm. Autophagy is a key instrument of cellular response to several stresses, including endoplasmic reticulum (ER) stress. Cancer cells have developed high dependency on autophagy to overcome the hostile tumor microenvironment. Thus, pharmacological activation or inhibition of autophagy is emerging as a novel antitumor strategy. ERK5 is a novel member of the MAP kinase family that is activated in response to growth factors and different forms of stress. Recent work has pointed ERK5 as a major player controlling cancer cell proliferation and survival. Therefore small-molecule inhibitors of ERK5 have shown promising therapeutic potential in different cancer models. Here, we report for the first time ERK5 as a negative regulator of autophagy. Thus, ERK5 inhibition or silencing induced autophagy in a panel of human cancer cell lines with different mutation patterns. As reported previously, ERK5 inhibitors (ERK5i) induced apoptotic cancer cell death. Importantly, we found that autophagy mediates the cytotoxic effect of ERK5i, since *ATG5*ˉ^/^ˉ autophagy-deficient cells viability was not affected by these compounds. Mechanistically, ERK5i stimulated autophagic flux independently of the canonical regulators AMPK or mTORC1. Moreover, ERK5 inhibition resulted in ER stress and activation of the Unfolded Protein Response (UPR) pathways. Specifically, ERK5i induced expression of the ER luminal chaperone BiP (a hallmark of ER stress), the UPR markers CHOP and ATF4, and the spliced form of XBP1. Pharmacological inhibition of UPR with chemical chaperone TUDC, or ATF4 silencing, resulted in impaired ERK5i-mediated UPR, autophagy and cytotoxicity. Overall, our results suggest that ERK5 inhibition induces autophagy-mediated cancer cell death by activating ER stress. Since ERK5 inhibition sensitizes cancer cells and tumors to chemotherapy, future work will determine the relevance of UPR and autophagy in the combined use of chemotherapy and ERK5i to tackle Cancer.

## Introduction

Macroautophagy, hereafter referred to as autophagy, is a highly conserved process that preserves cellular homeostasis by mediating the lysosomal degradation of cytoplasmic content (Levine and Kroemer, 2019). Mechanistically, autophagy involves the formation of transient double-membrane organelles called autophagosomes, which sequester portions of the cytoplasm and organelles, which are ultimately delivered to lysosomes for degradation (He and Klionsky, 2009). Autophagy is a key instrument of cellular response to several stresses, including nutrient starvation, hypoxia, protein aggregation and endoplasmic reticulum (ER) stress (Yin et al., 2016). Intensity and duration of the stimuli dictates the outcome of autophagy, and persistent stimulation of autophagy can lead to activation of cell death pathways, resulting in cytotoxic autophagy (Bialik et al., 2018). In cancer cells, autophagy plays a dual and paradoxical role in tumor suppression and tumor promotion (Singh et al., 2018). Since cancer cells can regulate autophagy as a response to cancer treatments, pharmacologic manipulation of autophagy represents a new strategy to design new anti-cancer therapies (Cirone et al., 2019). In this context, several antitumor molecules induce cancer cell death by modulating autophagy, including tetahydrocannabinol (Hernandez-Tiedra et al., 2016), resveratrol (Zhang et al., 2013) or ABTL0812 (Munoz-Guardiola et al., 2020). These molecules induce autophagy-mediated cancer cell death.

The endoplasmic reticulum (ER) develops an essential biosynthetic function and acts as a calcium reservoir, thus participating in cellular signalling. ER is the site of the cell where proteins and lipids are synthetized. Protein synthesis requires complex machinery, and it depends on the activity of molecular chaperones (such as BiP/GRP78 and GRP94), foldases (such as protein-disulphide isomerase, PPI) and quality control proteins (calnexin, calreticulin) to ensure proper folding and assembly of proteins (Brodsky and Skach, 2011). Different physiological and pathological conditions, including nutrient deprivation, hypoxia and viral infection, can affect the capacity of protein folding in the ER, leading to the accumulation of misfolded proteins within the ER lumen, a condition known as ER stress (Jain, 2017). In response to ER stress, a specific signalling network referred as the Unfolded Protein Response (UPR) is activated to reduce the load of unfolded proteins in the ER lumen. UPR restores cellular homeostasis by blocking general protein translation and activating a gene transcription programme directed to increase ER folding capacity (Hetz, 2012).

The UPR is controlled by three transmembrane ER stress protein sensors, namely ATF6 (activating transcription factor 6), IRE1 (inositol requiring enzyme 1) and PERK (PKR-like ER kinase), which in turn are controlled by the ER luminal chaperone BiP. Under basal conditions, BiP sterically represses the activity of these three sensors by binding their respective luminal domains. When ER homeostasis is perturbed, BiP dissociates from these sensors to bind accumulated unfolded proteins, allowing the homodimerization-mediated activation of PERK and IRE1, as well as translocation of ATF6 to Golgi where is activated by specific proteases (Walter and Ron, 2011). ATF6 and IRE1 axes induce transcription of the ER chaperones BiP and GRP94, proteins involved in ER-associated degradation (ERAD) of misfolded proteins, and of the XBP1 transcription factor (Calfon et al., 2002; Yamamoto et al., 2007). In turn, PERK promotes general protein translation arrest by phosphorylating and inactivating the initiation factor eIF2α (Liu et al., 2000). However, few specific proteins escape from this arrest and are upregulated, such as the ATF4 transcription factor that activates expression of proteins involved in protein folding, amino acid metabolism and autophagy (Schroder and Kaufman, 2005). In some circumstances, the adaptive responses provide by the UPR after a sustained ER stress might be insufficient to restore protein-folding homeostasis. In this scenario, unresolved ER stress promotes a UPR-mediated cell death programme that can be initiated by all three UPR sensors (Shore et al., 2011), being PERK-eIF2α-ATF4-CHOP the most studied pathway. Mechanistically, active ATF4 induces expression of CHOP (C/EBP homologous protein) transcription factor, which represses translation of the antiapoptotic Bcl-2 gene (McCullough et al., 2001) and activates expression of proapoptotic BIM (Puthalakath et al., 2007), among others.

Extracellular regulated kinase 5 (ERK5) is the most recently discovered member of the mitogen-activated protein kinase (MAPK) family, and is activated in response to growth factors and to different forms of cellular stress (Kato et al., 1997; Kato et al., 1998). ERK5 is activated by direct phosphorylation of the kinase domain by the MAPK kinase 5 (MEK5) (Zhou et al., 1995). Upon activation of the pathway, MEK5 phosphorylates cytosolic ERK5 to drive its translocation to the nucleus, where active ERK5 acts as a transcriptional activator (Kasler et al., 2000). Thereby, ERK5 has been reported to promote cell proliferation and cell cycle progression, among others. There are increasing evidence showing that ERK5 plays an important role in cancer cell proliferation and survival. Thus, ERK5 silencing or pharmacological inhibition compromises viability of numerous cancer cell lines, as well as impairs tumor growth in xenograft models (Reviewed in Gomez et al. (2016), Pereira and Rodrigues (2020), Stecca and Rovida (2019)).

During the last years, several ERK5 small molecule inhibitors (ERK5i) have been developed. In all the cases, pharmacological inhibition of ERK5 resulted in activation of apoptosis in a broad number of human cancer cell lines (Hoang et al., 2017). Here, we provide evidence that ERK5 signaling pathway acts a negative regulator of autophagy in cancer cells. Furthermore, ERK5 inhibition or silencing resulted in autophagy-mediated apoptotic cancer cell death. We also provide evidence that ER stress and UPR mediated in ERK5i-induced cytotoxic autophagy. Our results underline the role of autophagy in the anticancer potential of ERK5 inhibitors.

## Materials and Methods

### Cell Culture and Cell Treatment

Human cervical HeLa and pancreatic ductal MiaPaCa-2 adenocarcinoma cell lines were obtained from the American Collection of Cell Cultures (ATCC). Endometrial adenocarcinoma Ishikawa cells were from European Collection of Authenticated Cell Cultures (ECACC). MEF cells obtained from wild type or ATG5 deficient (ATG5^-/-^) mice and immortalized by T-SV40 virus infection (Salazar et al., 2009) were kindly provided by Dr. G. Velasco (Complutense University, Spain). HeLa, MiaPaCa-2, and MEF cells were maintained in Dulbecco’s modified Eagle’s medium (DMEM, ThermoFisher) supplemented with 10% foetal bovine serum (FBS, Gibco) and 1% Penicillin/Streptomycin (Gibco). Ishikawa cells were maintained in Minimal essential medium (MEM, Sigma) containing 5% FBS, 1% Penicillin/Streptomycin and 1% L-Glutamine (Gibco). Cells were maintained at 37°C in a humidified atmosphere containing 5% CO_2_.

Cells were plated at the desired confluence and allowed to attach the plate for 24 h. Treatments were performed for the indicated times and concentrations, with complete medium or starvation medium (culture medium containing 0.5% FBS), as indicated for each experiment. ERK5 inhibitors JWG-071 (in-house synthesized), XMD8-92 (Tocris) and AX15836 (MedChemExpress), or MEK5 inhibitors BIX 02188 and BIX 02189 (Selleckchem) were diluted in dimethyl sulfoxide (DMSO, Sigma). For monitoring the autophagic flux, cells were pre-incubated with a combination of lysosomal proteases inhibitors E64d (Sigma-Aldrich) and Pepstatin A (PA, Sigma-Aldrich) 2 h before treatment with JWG-071. ER stress mitigation with chemical chaperones was performed with sodium tauroursodeoxycholate (Sigma-Aldrich) dissolved in distilled water. Brefeldin A (Sigma-Aldrich) was diluted in absolute ethanol.

### Cell Lysis and Protein Quantitation

Cells were lysed in ice-cold radioimmunoprecipitation assay (RIPA) buffer (25 mM Tris-HCl pH 7.9; 150 mM NaCl; 1 mM EGTA; 5 mM sodium pyrophosphate; 0.5% (w/v) deoxycholic acid; 0.1% (w/v) sodium dodecyl sulphate (SDS); 1% (w/v) NP-40). Cell lysates were sonicated and centrifuged at 12,000 rpm for 12 min at 4^o^C. Soluble fractions were then transferred to a new eppendorf tube and kept at −20°C until use. Protein quantification was performed by the Bradford assay Coomassie Blue G-250 (Pierce) dye, using BSA (Sigma) as a standard. Absorbance was measured at 595 nm in a LKB spectrophotometer.

### DNA Transfection and DNA Constructs

Cells were transfected with Lipofectamine^TM^ 2000 (Life Technologies), as described before (Erazo et al., 2013). Optimal DNA:Lipofectamine ratio (w:v) was determined before performing transfections. Briefly, Lipofectamine^TM^ 2000 and DNA were diluted in OptiMEM medium (Gibco) in separate tubes, vortexed and added to the plasmid-containing solution. Transfection solution was added to cells and, after 4-h incubation, the medium was changed and transfected cells were left 48 h. pEGFP-C1 plasmid encoding for GFP-tagged human LC3 was from Dr. Guillermo Velasco (Universidad Complutense Madrid, Spain).

### Transfection of siRNAs

Control siRNA (5ʹ-GUA​AGA​CAC​GAC​UUA​UCG​C-3ʹ) and ATF4-directed siRNA (ATF4-1: 5ʹ-GCC​UAG​GUC​UCU​UAG​AUG​A-3ʹ) were purchased from Sigma-Aldrich. siRNAs were transfected (Lipofectamine^TM^ 2000) in cells, and cellular lysates were obtained 48 h post-transfection.

### Lentivirus Infection and shRNA-Mediated ERK5 Silencing

Lentiviral vector encoding ERK5-directed shRNAs (TRCN0000197264/pLKO.1, seq. CCG​GGT​TCA​TCT​CAG​ACC​CAC​CTT​TCT​CGA​GAA​AGG​TGG​GTC​TGA​GAT​GAA​CTT​TTT​TG) was from Sigma. Control lentiviral particles were generated using an empty pLKO.1 plasmid. Lentiviral particles were generated in HEK-293 cells by co-transfecting the pLKO.1 vector together with the packaging vector psPAX2 and the viral envelope vector pMD2G, as described before (Erazo et al., 2020). MiaPaCa-2 cells (40% density) were infected with lentiviral shRNA particles, and 24 h post-infection, medium was changed. After 96 h, cells were lysed with RIPA buffer, and stored at −20°C until use.

### Cell Viability Assays

Cell viability was determined using the tetrazolium dye 3-(4,5-dimethylthiazol-2-yl)-2,5-diphenyltetrazolium bromide (MTT, Sigma) reduction assay, as described before (Erazo et al., 2016). MTT absorbance was measured in a Synergy HT microplate reader (BioTek Laboratories, Inc.) at 560 nm reading wavelength and 620 nm reference wavelength. Each treatment was performed by tetraplicates. Cell viability was also determined using a LIVE/DEAD viability/cytotoxicity kit assay (ThermoFisher).

### Analysis of Apoptosis by Flow Cytometry: Nuclear Staining and Flow Cytometry

Cells were seeded and treated with JWG-071 for 48 h. Then, cells were trypsinized, washed sequentially with PBS and binding buffer, and incubated in binding buffer with Annexin V and/or propidium iodide (Invitrogen) for 15 min (protected from the light). Samples were analyzed in a Beckman Coulter FC 500 flow cytometer.

### Electrophoresis and Immunoblot Analysis

Proteins were resolved by SDS-polyacrylamide gel electrophoresis, using the Mini-Protean system (Bio-Rad). Samples were diluted in loading buffer (25 mM Tris-HCl pH 6.8; 2% (w/v) SDS; 10% glycerol; 0.002% (w/v); 5% (v/v) β-mercaptoethanol) and heated at 97°C for 5 min. 10–40 µg of each sample were loaded per well. Electrophoresis was run at a constant voltage of 130 V for 80 min in electrophoresis buffer (25 mM Tris; 192 mM glycine; 20 (w/v) SDS). Then, proteins were transferred onto a 0.45 µm nitrocellulose membrane (Schleicher and Schurrell) using a Mini Trans-Blot Electrophoresis Transfer Cell (Bio-Rad) and Tris-Glycine buffer (25 mM Tris, 192 mM glycine, 20% (v/v) methanol). Membranes were blocked with TBS-Tween buffer (20 mM Tris-HCl, pH 7.6, 150 mM NaCl, 0.2 (v/v) Tween) containing 5% (w/v) non-fat milk or 5% (w/v) bovine serum albumin (BSA), incubated 16 h with the corresponding primary antibody, washed and incubated with the appropriated peroxidase-conjugated secondary antibody (Pierce). Protein detection was performed by chemiluminescence, using the Clarity^TM^ ECL Western Blotting Substrate kit (Bio-Rad) and photographic films (Fuji Medical X-ray film; Fujifilm). The following primary antibodies were used: AMPK (CST # 5832, 1:10,000), ATG5 (Abcam #ab109490, 1:1,000), ATF4 (CST # 11,815, 1:1,000), β-Actin (Santa Cruz, # sc-47778, 1:4,000), BiP (CST # 53,177, 1:2,000), CHOP (CST # 5554 1:500), Cleaved caspase 3 (CST # 9661, 1:500), ERK5 (CST # 3372, 1:1,000), GAPDH (Invitrogen # AM4300, 1:150,000), Hsp90 (CST # 4874, 1:10,000), LC3B (Abcam, # ab48394, 1:10,000), MEK5 (Santa Cruz # sc-365119), p62 (Enzo Life Sciences # BML-PW9860, 1:10,000), pT172-AMPK (CST # 2535, 1:1,000), pS235/236-S6 (CST # 4858, 1:40,000), S6 (CST # 2317, 1:40,000), pS555-ULK (CST # 5869, 1:1,000), pS757-ULK (CST # 6888, 1:1,000), ULK (CST # 8054, 1:1,000).

### Fluorescence Confocal Microscopy

HeLa cells were transfected with a vector encoding for GFP-tagged LC3. Forty eight hours later, cells were treated with JWG-071 or vehicle, and incubated for the indicated times. Cells were fixed with a solution containing 4% formaldehyde (Electron Microscopy Sciences), 0.1% NP40 (Merck-Sigma), 1 μg/ml Hoechst 33342 (ThermoFisher) diluted in PBS. Cells were observed in a confocal fluorescence microscope (Zeiss LSM 700). Images of multiple fields were acquired for each treatment, and analyzed with ImageJ software (NIH, United States).

### RNA Extraction From Cells. Maxwell

Total RNA was extracted from cells using the Maxwell^®^ RSC simplyRNA Cells Kit (Promega). Genomic DNA was digested with DNase, according to the manufacturer’s instructions. Extracted RNA was retrotranscribed to cDNA, and qRT-PCR was performed using TaqMan^TM^ Universal PCR Master Mix and the following TaqMan^TM^ probes spanning at exon junctions (Applied Biosystems): human *ATF4* (Hs00909569_g1), human *DDIT3/CHOP* (Hs99999172_m1), human *GAPDH* (Hs03929097_g1). For analysis of XBP1 splicing, qRT-PCR was performed using SYBR™ Green PCR Master Mix and the specific primers for the spliced human *XBP1* (forward 5′-CTG​AGT​CCG​CAG​CAG​GTG​CA-3′, reverse 5′-GGT​CCA​AGT​TGT​CCA​GAA​TGC​CCA​A-3′), as well as for human *GAPDH* (forward 5′-CAA​ATT​CCA​TGG​CAC​CGT​CA-3′, reverse 5′-GAC​TCC​ACG​ACG​TAC​TCA​GC-3′). Values were normalized to *GADPH* (*ATF4* and *CHOP*) or *HPRT1* (*XBP1s*) levels. Relative expression levels were determined using the 2^−ΔΔCt^ method.

### Statistical Analysis and Figure Generation

Graphics and statistical analyses were generated using GraphPad Prism 8.0.1 software. Figures were generated using Adobe Photoshop software. All *in vitro* data were assessed using one-way ANOVA followed by Tukey multiple comparison test or Student’s *t*-test. Statistical significance cut-off was established as P < 0.05. Significance values are expressed as *p < 0.05; **p < 0.01; ***p < 0.001; ****p < 0.0001. Data in the figures are presented as mean ± SD, with the result of the statistical test. Synergism analysis was performed using the Compusyn software (Chou, 2010).

## Results

### Inhibition of MEK5/ERK5 Pathway Induces Autophagy in Cancer Cells

Several studies have shown a key role of oncogenic signaling pathways, such as Ras/Raf/ERK and PI3K-mTOR, in regulating autophagy in cancer cells (Zada et al., 2021). To investigate whether ERK5 modulates autophagy in cancer cells, we used three cancer cell lines that show different oncogenic mutations: pancreatic ductal adenocarcinoma MiaPaCa-2 cells (containing mutated KRAS), endometrial adenocarcinoma Ishikawa cells (PTEN null), and cervical carcinoma HeLa cells (no alteration in Ras/Raf/ERK or PI3K-mTOR pathways). To do so, we first checked the effect of the new specific ERK5 inhibitor JWG-071, a small compound that do not show off-target activity on bromodomain-containing proteins (BDRs) (Wang et al., 2018).

**FIGURE 1:**
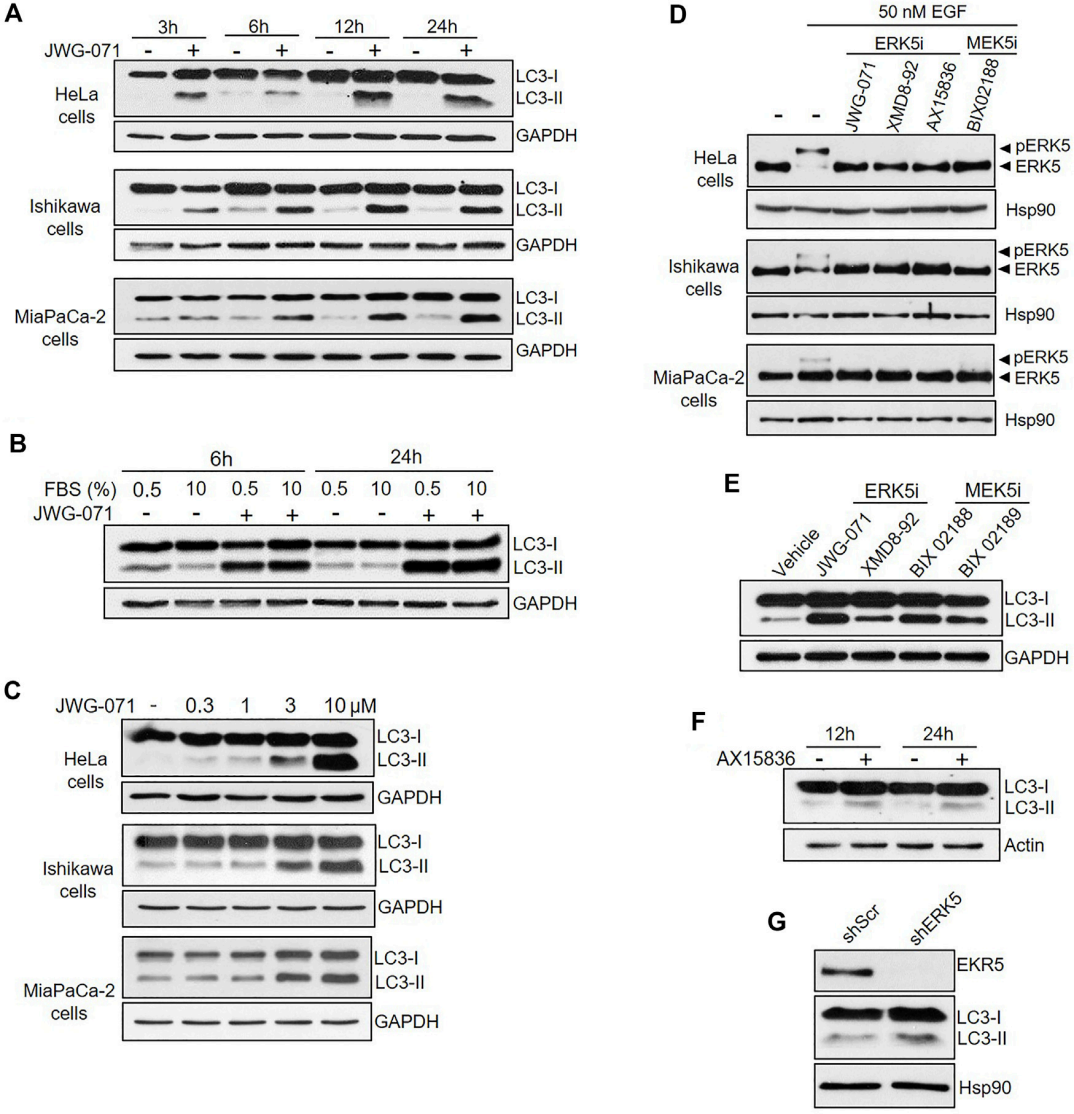
Inhibition of MEK5/ERK5 pathway induces autophagy in serum starved and non-starved Cancer cells. **(A,B)** ERK5 inhibition induces sustained autophagy. Cells under serum starvation (0.5% FBS), **(A)** or cultured with 10% FBS (**B**, Hela Cells) were treated with vehicle (DMSO) or 10 μM JWG-071 for the indicated times. Cells were lysed and levels of the autophagy-marker protein LC3 were visualized by immunoblotting. GAPDH levels are shown as a loading control. Blots are representative of three independent experiments. **(C)** ERK5 inhibition stimulates autophagy in a dose-dependent manner. Serum-starved cells were treated with DMSO or the indicated concentrations of JWG-071 for 24 h, and cell lysates were analysed by immunoblot as in **(A,B)**. Blots are representative of two separate experiments. **(D)** ERK5 or MEK5 inhibition impairs cellular ERK5 activity. Serum-starved cells were treated with the indicated inhibitors (5 μM), before incubation with 50 nM EGF for 30 min. Blots are representative of two separate experiments. **(E)** Inhibition of the MEK5/ERK5 pathway actives autophagy. Ishikawa cells were treated with either ERK5 inhibitors (5 μM JWG-071 or 10 μM XMD8-92) or MEK5 inhibitors (10 μM BIX 02188 or 10 μM BIX 02189) for 24 h. Controls were treated with vehicle. Autophagy was detected by immunoblotting as in **(A,B)**. GAPDH levels are shown as a loading control. **(F)** ERK5i AX15836 induces autophagy. MiaPaCa-2 cells were treated with 3 μM AX15836 for 12 or 24 h and autophagy was monitored as in **(A,B)**. Actin levels are shown as a loading control. **(G)** ERK5 silencing induces autophagy. MiaPaCa-2 cells were infected with lentiviral particles encoding for shRNA sequence to target ERK5. Levels of indicated proteins were detected by immunoblot analysis.

One of the hallmarks of autophagy is the conversion of the soluble form of LC3 (MAP1LC3B, also called Atg8) to lipidated LC3, known as LC3-II (MAP1LC3B-II), which is associated to autophagosomes (Klionsky et al., 2021). When autophagy is induced, the soluble form of the protein LC3 (hereafter, LC3-I) undergoes covalent attachment of a molecule of phosphatidylethanolamine, resulting in association of the lipidated form LC3-II to autophagosomal membranes. Lipidated LC3-II can be differentiated from LC3-I by immunoblotting, since it has a faster electrophoretic mobility on SDS/PAGE gels (Kabeya et al., 2004). Human cervical (HeLa), endometrial (Ishikawa) and pancreatic (MiaPaCa-2) adenocarcinoma cell lines were serum starved and treated with the ERK5 inhibitor JWG-071 for different times, and LC3-I/LC3-II levels were evaluated by immunoblotting. Inhibition of ERK5 resulted in increased levels of the lipidated form LC3-II in the three cancer cell lines tested (Figure 1A), indicative of either enhanced autophagy or a block in autophagy. The increase of LC3 lipidation was observed at 3 h of incubation with JWG-071, and it was sustained for at least 24 h, suggesting that ERK5 inhibition exacerbates the autophagy induced by serum deprivation (Figure 1A). We next investigated the effect of ERK5 inhibition in cells cultured with serum (10% FBS). In these conditions, ERK5 inhibition increased LC3-II levels at a similar extent than in serum starved cultures (Figure 1B), indicating that ERK5 inhibition induces LC3-II accumulation independently of serum deprivation. JWG-071 titration experiments further demonstrated a dose-dependent effect on LC3-II accumulation in the three cell lines tested, which was significantly observed at 1–3 μM concentration of the ERK5 inhibitor (Figure 1C).

**FIGURE 2:**
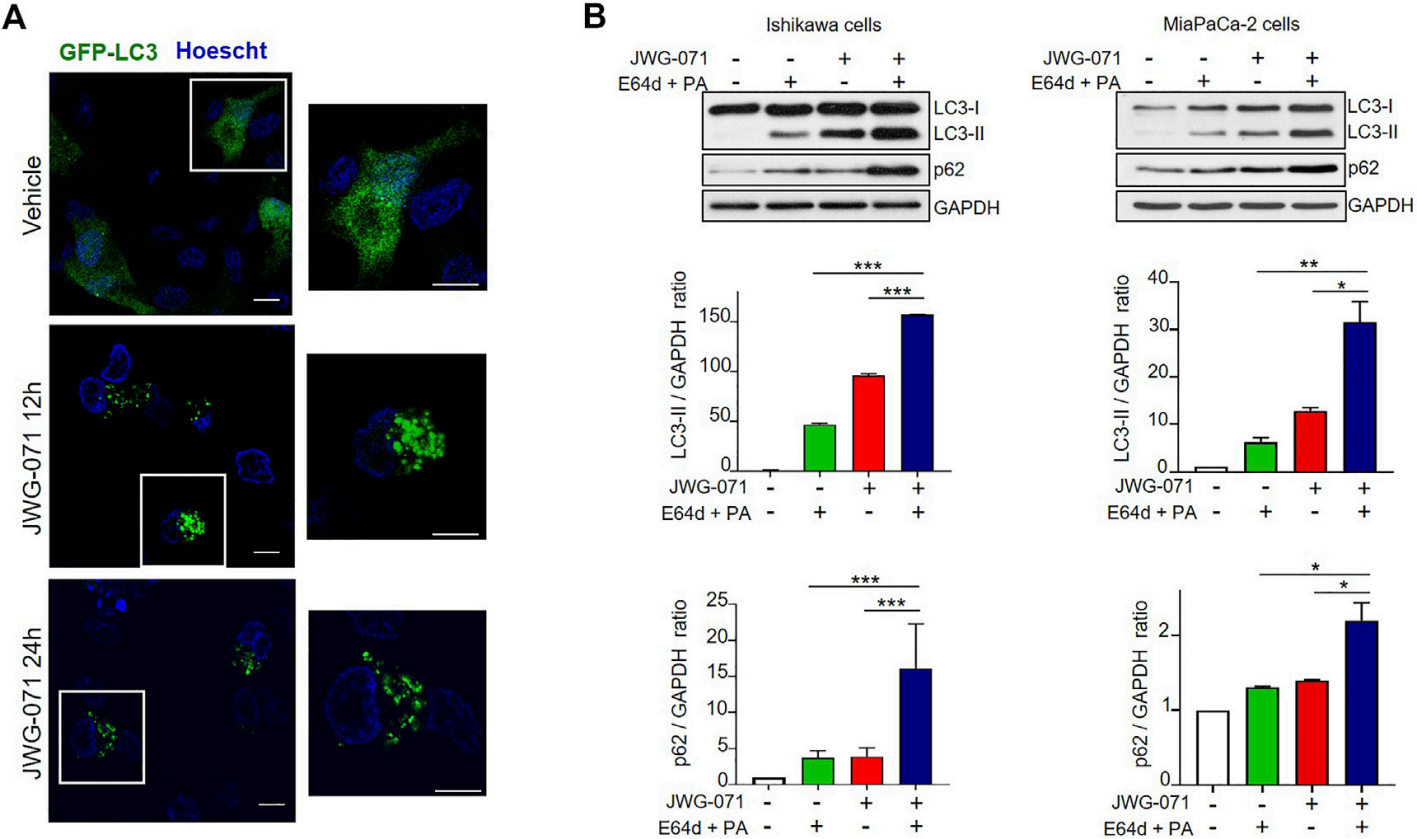
ERK5 inhibition induces autophagic flux. **(A)** ERK5 inhibition induces LC3 association with autophagosomes. HeLa cells transiently expressing GFP-LC3 (green) were treated with vehicle or 5 μM JWG-071 for 24 h. After staining nuclei *in vivo* with Hoechst 33342 (blue), cells were fixated and visualized by confocal fluorescence microscopy. Punctuate represents autophagosome formation. Similar results were obtained in two independent experiments. Scale bar. 10 μm. **(B)** ERK5 inhibition induces autophagic flux. Ishikawa and MiaPaCa-2 cells were pre-incubated for 2 h with ethanol (vehicle) or a combination of lysosomal proteases inhibitors E64d (10 μM) and Pepstatin-A (PA, 10 mg/ml) before treatment with 5 μM JWG-071 or DMSO for further 24 h. Cell lysates were probed against autophagy markers LC3 and p62. GAPDH levels were assessed as a loading control. Similar results were obtained in two separate experiments. Histograms show the quantification of LC3-II levels relative to GAPDH, estimated by densitometric analysis. Values represent mean ± SD of two different determinations. *p < 0.05 **p < 0.01, ***p < 0.001 from treatment with E64d + PA alone or JWG-071 alone.

In order to preclude any potential JWG-071 off-target effect, we explored whether a battery of ERK5 or MEK5 inhibitors induce LC3 lipidation. We used the ERK5 inhibitor XMD8-92 (which has BRD4 activity, (Yang et al., 2010)), the more recently developed AX15836 (Lin et al., 2016), and the upstream kinase MEK5 inhibitor BIX02188 (Razumovskaya et al., 2011). All inhibitors blocked activation of ERK5 in response to EGF, as shown by immunoblot analysis (active ERK5 autophosphorylates resulting in a slower migrating band) (Figure 1D)**.** As expected, ERK5/MEK5 inhibitors enhanced LC3 lipidation (Figures 1E,F). This observation was furtherly assessed using lentiviral shRNA specific for ERK5. ERK5 silencing also resulted in increased LC3-II levels (Figure 1G), suggesting that attenuation of the MEK5/ERK5 pathway might result in increased cellular autophagy. This was confirmed by confocal fluorescence microscopy, using HeLa cells that transiently overexpressed GFP-tagged LC3. As expected, LC3 showed a diffuse, homogeneous localization throughout the cytoplasm and nucleus in resting cells, whereas JWG-071 induced a punctuate pattern for LC3, indicative of association to autophagosomes (Figure 2A; Klionsky et al., 2021).

Finally, to demonstrate that the increased LC3-II expression observed in response to ERK5 inhibition was due to activation of autophagy, we investigated the effect of ERK5i on the autophagic flux. Autophagic flux comprises from autophagosome assembly upon induction until its fusion with a lysosome, where autophagic cargos are degraded (Klionsky and Ohsumi, 1999; Glick et al., 2010). Given that autophagosome-lysosome fusion is subjected to independent regulation, an accumulation of autophagosomes does not necessarily indicate a higher level of autophagy. For instance, a block in autophagy can result in accumulation of LC3-II and of autophagosomes. Hence, it is imperative to evaluate the autophagic flux to confirm that autophagy is induced. To this end, we co-treated Ishikawa or MiaPaCa-2 cells with JWG-071 and a combination of E64d and Pepstatin-A (PA) lysosomal protease inhibitors that block the final step of autolysosomal degradation. Treatment with both protease inhibitors resulted in a significant accumulation of LC3-II and the autophagic cargo protein p62 (Figure 2B), indicating that ERK5 inhibition induces dynamic autophagy in cancer cells.

### ERK5 Inhibition Induces Autophagy-Mediated Cancer Cell Death

**FIGURE 3:**
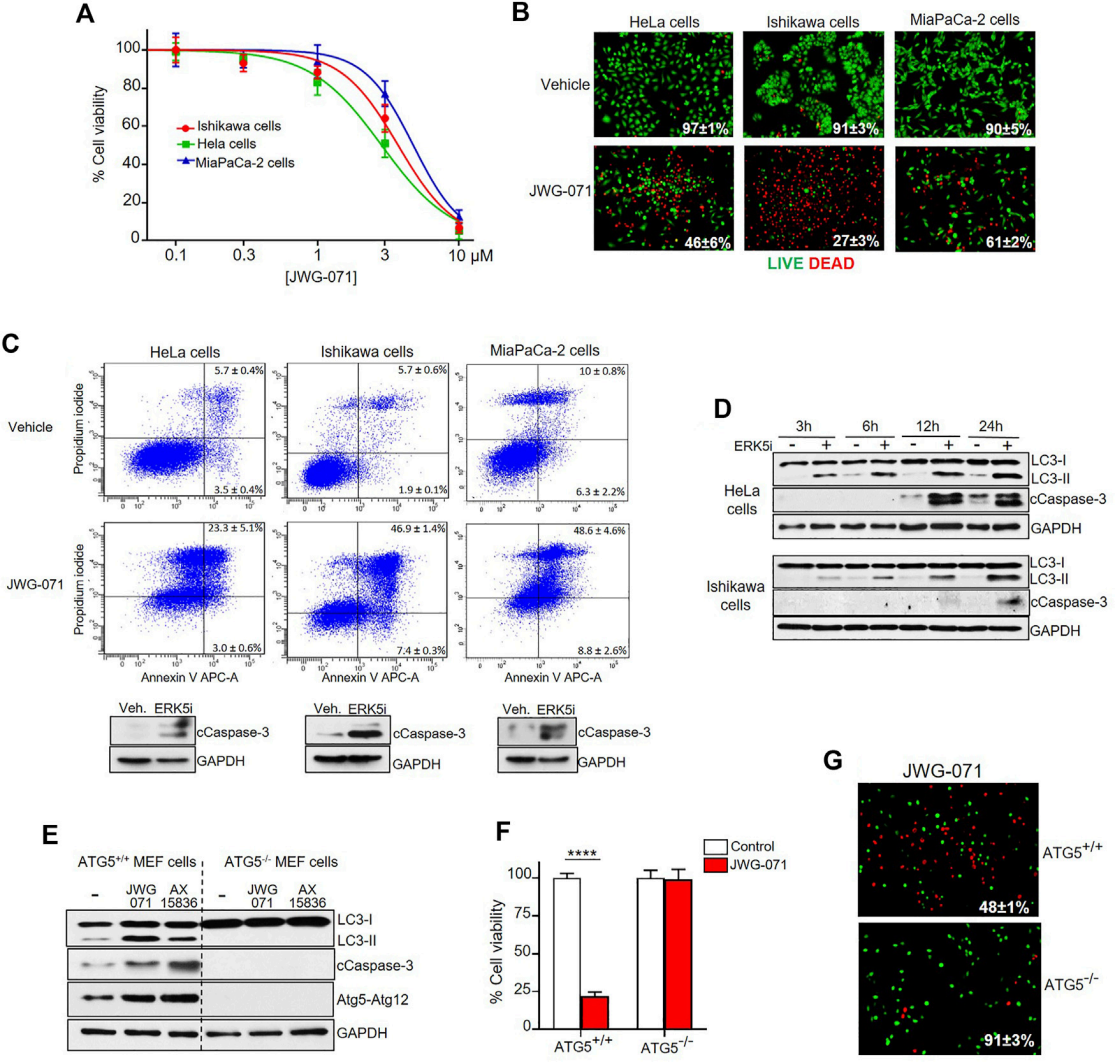
ERK5 inhibition induces autophagy-mediated apoptotic cancer cell death. **(A)** ERK5i induces cytotoxicity. MTT cytotoxicity assay in a panel of human Cancer cells. JWG-071 was incubated for 48 h at the indicated concentrations. Values represent mean ± SD of three independent experiments, each performed in tetraplicates. **(B)** ERK5i induces cell death. Cells were treated with 10 μM JWG-071 for 36 h, stained with LIVE/DEAD reagent, and alive (green) and dead (red) cells were visualized by fluorescence microscopy. Figure shows representative fields, and percetage of alive cells is given. **(C)** ERK5i induces apoptotic cancer cell death. Percentage of apoptotic cells was determined by flow cytometry (Annexin V/Propidium iodide staining) at 48 h following treatment with 5 μM JWG-071. Representative flow cytometry plots of cells are shown. Similar results were obtained in three independent experiments, each performed in duplicates. Immunoblot panels show active caspase-3 (cleaved caspase-3 levels, cCaspase 3) in response to JWG-071. **(D)** Autophagy induced by ERK5i precedes caspase-3 fragmentation. HeLa and Ishikawa cells were treated with vehicle or 5 μM JWG-071 for the indicated times, and autophagy (LC3) and apoptosis (Cleaved Caspase-3, cCaspase-3) markers were monitored by immunoblot. GAPDH was used as a loading control. **(E)** ERK5i does not activate caspase-3 in autophagy-deficient transformed MEF cells. WT or ATG5^−/−^ transformed MEF cells were treated with vehicle or 5 μM JWG- 071 for the indicated times, and levels of autophagy and apoptosis were monitored by immunoblotting of LC3 and cleaved caspase-3, respectively. GAPDH was used as loading control. Results are representative of three independent experiments. **(F,G)** Autophagy-deficient ATG5^−/−^ transformed MEF cells are insensitive to ERK5 inhibition. **(F)** Wild type or ATG5^-/-^ MEFs were serum starved and treated with either vehicle (white columns) or 10 μM JWG-071 (red columns) for 48 h. Cell viability was assessed by MTT assay. Values (mean ± SD) are representative of three separate experiments, each performed in tetraplicates. ****p < 0.0001 from WT MEF cells. **(G)** Wild type or ATG5^-/-^ MEFs were serum starved, treated with 10 μM JWG-071 or 48 h, stained with LIVE/DEAD reagent, and alive (green) and dead (red) cells were visualized by fluorescence microscopy. Figure shows representative fields, and percetage of alive cells is given.

Next, we evaluated the effect of ERK5 inhibition in the viability of HeLa, Ishikawa and MiaPaCa-2 cancer cells. Cell viability (MTT) assays showed that JWG-071 decreased the viability in a concentration-dependent manner in all three cancer cell lines tested, with IC_50_ values ranking 3–6 μM (Figure 3A). Impaired cell viability in response to ERK5i was confirmed using live/dead assay (Figure 3B). As reported for other ERK5 inhibitors (Pereira and Rodrigues, 2020), JWG-071 treatment resulted in apoptotic cell death, as determined by flow cytometry analysis of Annexin V/Propidium iodide staining (Figure 3C). Immunoblot analysis of active/cleaved caspase 3 confirmed that JWG-071 induced apoptosis in the three cancer cell lines tested (Figure 3C).

Over the last years, several autophagy-activating small molecules have shown anticancer activity (Salazar et al., 2009; Puissant et al., 2010; Erazo et al., 2016; Hernandez-Tiedra et al., 2016). These compounds induce the so-called autophagy-mediated cell death, which ultimately leads to activation of apoptotic or necrotic cell death (Bialik et al., 2018). Given that ERK5 inhibitors show antitumor activity *in vitro* (cell lines) and *in vivo* (xenograft models) by inducing apoptosis (see Gomez et al. (2016), Pereira et al. (2016), for review), we next inspected whether autophagy was involved in ERK5 inhibition-mediated cytotoxicity. Interestingly, we observed that autophagy induced by JWG-071 preceded caspase-3 cleavage in HeLa and Ishikawa cancer cells (Figure 3D).

To investigate the role of autophagy in the apoptosis induced by ERK5 inhibition, we employed oncogene-transformed MEF (mouse embryonic fibroblast) cells derived from ATG5^-/-^ mice (Salazar et al., 2009). Atg5 is an essential protein for autophagosome formation, given its critical role in the extension of the phagophoric membrane (Virgin and Levine, 2009). Therefore, ATG5^-/-^ cells are deficient for autophagy. As expected, ERK5 inhibition by JWG-071 or AX15836 induced LC3 lipidation (autophagy) in immortalized ATG5^+/+^ MEF cells, but not in autophagy-deficient ATG5^−/−^ MEF cells. More importantly, autophagy-deficient ATG5^−/−^ MEF cells did not show active caspase-3 in response to ERK5 inhibition, as observed for ATG5^+/+^ MEF cells (Figure 3E **)**. Of note, JWG-071 induced a significant increase on levels of Atg5-Atg12 conjugate in ATG5^+/+^ MEF cells. Since covalently binding of Atg12 to Atg5 is necessary for the formation of autophagosome (Romanov et al., 2012), this result provides a further evidence that ERK5i induces cellular autophagy. Cell viability (MTT) and LIVE/DEAD assays showed that ATG5^−/−^ MEF cells were resistant to JWG-071-induced cell death. In contrast, with ATG5^+/+^ MEF cells showed elevated cytotoxicity in response to the ERK5i (Figures 3F,G **)**. Together, our results support the notion that ERK5 inhibition induces autophagy-mediated apoptosis in cancer cells.

### ERK5 Inhibition Induces ULK1-Independent Autophagy

**FIGURE 4:**
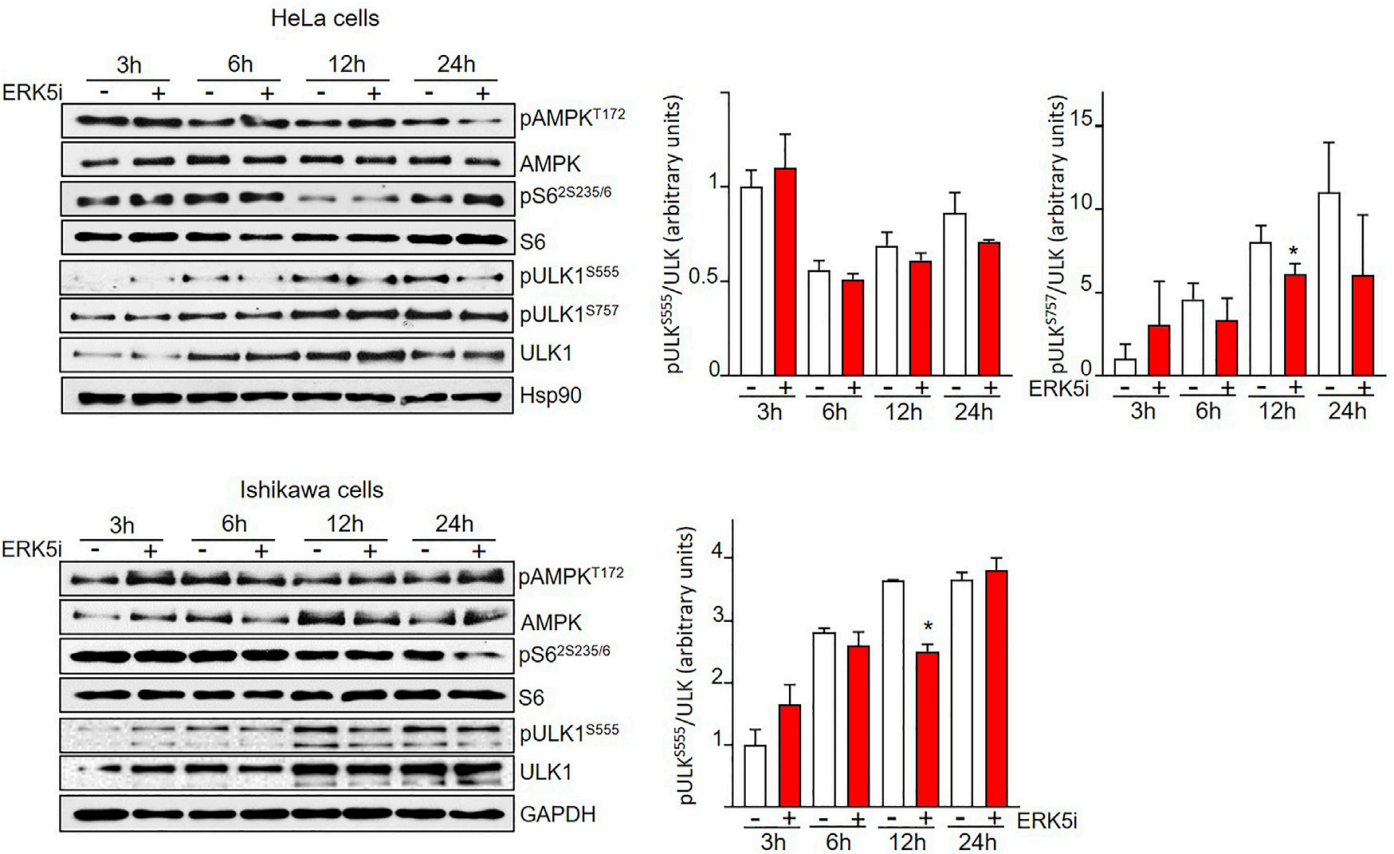
ERK5 inhibition induces autophagy independently of canonical ULK phosphorylation. HeLa and Ishikawa cells were treated for the indicated times with vehicle or 10 μM JWG-071. AMPK and mTORC1 pathways were analysed by immunoblotting, using total and phosphospecific antibodies (pThr172-AMPK and pSer235/236-S6). Total ULK and phospho-ULK (pSer666-ULK and pSer757-ULK) were also evaluated by immunoblotting. GAPDH is shown as a loading control. Blots are representative of at least three separate experiments. Right histograms show the quantification of pULK^Ser555^ and pULK^Ser555^ levels, relative to total ULK, estimated by densitometry. Values represent mean ± SD of two different determinations.

Two of the main canonical regulators of autophagy initiation are AMPK and mTORC1. Both protein kinases fine-tune autophagy by direct phosphorylation of ULK1/Atg1, the autophagy master regulator that coordinates autophagy levels by integrating various stress inputs (Zachari and Ganley, 2017). AMPK induces autophagy by direct phosphorylation of ULK1 at Ser317, Ser555, and Ser777. On the contrary, mTORC1 phosphorylation of ULK1 at Ser637 and Ser757 results in impaired autophagy (Kim et al., 2011). Therefore, we next interrogated the implication of AMPK and mTORC1 in the ERK5-mediated autophagy. To this end, we treated HeLa and Ishikawa cells with the ERK5 inhibitor JWG-071 for different times, and we used immunoblot analysis to study the activity of AMPK (by using the phosphospecific antibody anti-pThr172) and of mTORC1 (by monitoring levels of phosphorylated ribosomal protein S6). Neither Ishikawa nor HeLa cancer cells displayed significant changes in AMPK or mTORC1 activities in response to JWG-071, indicating that both pathways remained unaffected by ERK5 inhibition (Figure 4). We also analyzed ULK1 phosphorylation at residues Ser555 (AMPK) and Ser757 (mTORC1). Consistent with unaltered AMPK and mTORC1 activities, ULK phosphorylation status at Ser555 and Ser757 was not affected by ERK5 inhibition (Figure 4). Together, these results indicate that ERK5 inhibition induces autophagy independently of AMPK- or mTOCRC1-mediated ULK1 phosphorylation.

### ER Stress and UPR Mediate Autophagy Induced by ERK5 Inhibition

Endoplasmic reticulum (ER) stress and the subsequent activation of the Unfolded Protein Response (UPR) have been reported to induce cytotoxic autophagy in cancer cells under specific conditions (Salazar et al., 2009; Rashid et al., 2015). We hypothesized that ERK5 involvement on autophagy could be mediated by activation of the UPR in response to ER stress. The UPR relies on a specific signaling network that is controlled by different transmembrane ER stress protein sensors, namely IRE1/ERN1 (inositol requiring enzyme 1), PERK/EIF2AK3 (eukaryotic translation initiation factor 2-alpha kinase 3) and ATF6 (activating transcription factor 6) (Walter and Ron, 2011). These three sensors are controlled by the ER luminal chaperone BIP (also called HSPA5/GRP78). Under basal conditions, BiP sterically represses the activity of these three sensors by binding their respective luminal domains. When ER homeostasis is perturbed, BiP dissociates from these sensors to bind accumulated unfolded proteins, allowing the homodimerization-mediated activation of PERK and IRE1, as well as translocation of ATF6 to Golgi where is activated by specific proteases (Walter and Ron, 2011). Once activated, PERK allows activation of the eIF2a-ATF4-CHOP branch of the UPR, which promotes general protein translation arrest by phosphorylating and inactivating the initiation factor eIF2α (Liu et al., 2000). However, few specific proteins escape from this arrest and are upregulated, such as the ATF4 transcription factor that activates of proteins involved in protein folding, amino acid metabolism and autophagy (Schroder and Kaufman, 2005).

**FIGURE 5:**
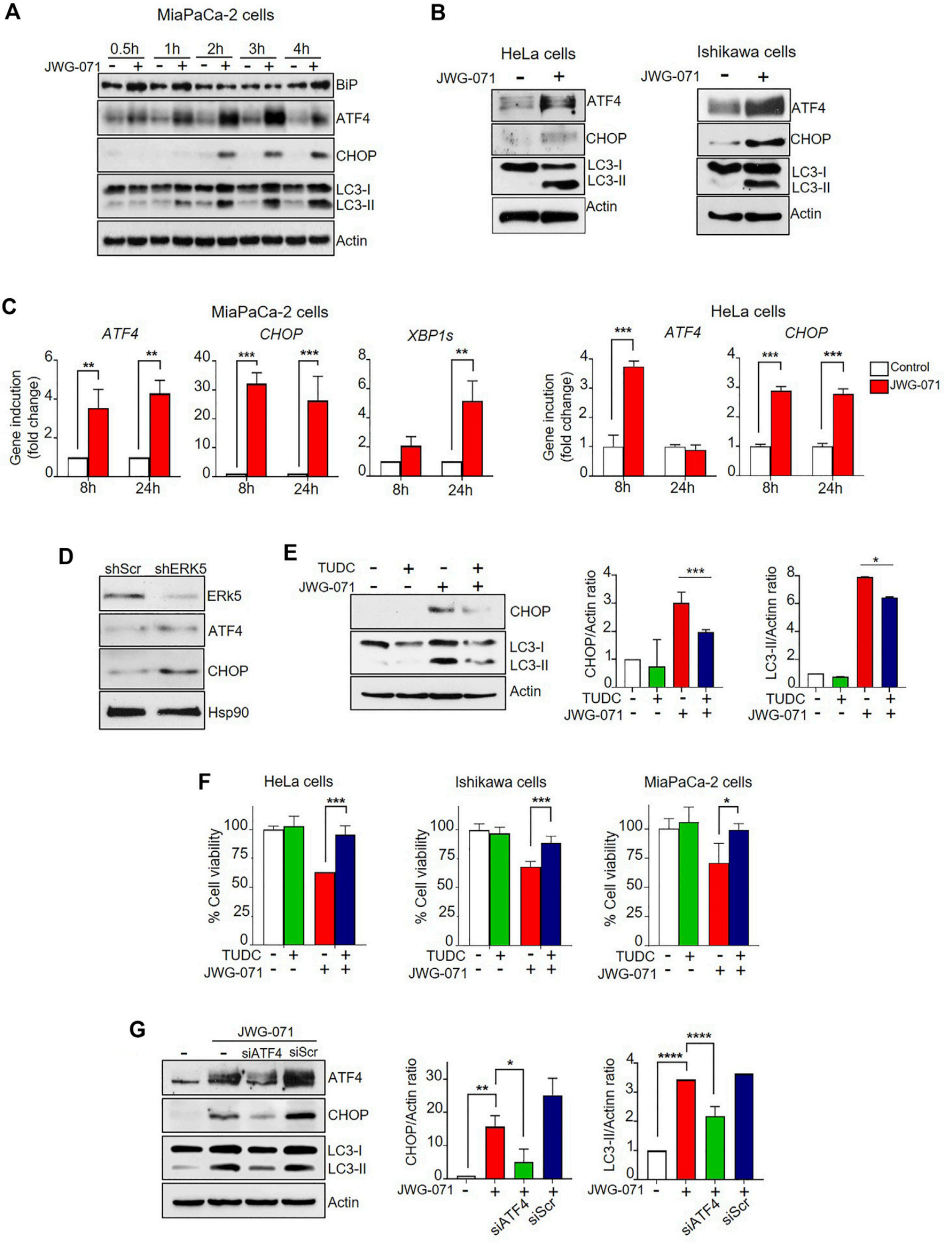
ER stress and UPR mediates ERK5i-induced autophagy. **(A)** ERK5i induces ER stress and UPR, which precedes autophagy. MiaPaCa-2 cells were treated for the indicated times with either vehicle or 5 μM JWG-071. The levels of the indicated proteins were monitored by immunoblot analysis. Actin was used as a loading control. **(B)** ERK5i induces UPR in HeLa and Ishikawa cells. Cells were treated with either vehicle or 5 μM JWG-071 for 24 h, and levels of the indicated proteins were monitored by immunoblot. **(C)** ERK5i induces gene expression of UPR markers. MiaPaCa-2 and HeLa cells were treated with vehicle or 5 μM JWG-071 for 8 or 24 h, and total RNA was extracted and retrotranscribed to cDNA. *ATF4*, *CHOP*, and spliced *XBP1* (*XBP1s*) mRNA levels were analyzed by qRT-PCR and normalized by *TBP* (*ATF4* and *CHOP*) or *HPRT1* mRNA levels (*XBP1s*). Values represent the mean ± SD of two separate experiments, each performed in duplicates. **p < 0.01 ***p < 0.001 from untreated cells. **(D)** ERK5 silencing induces UPR markers. MiaPaCa-2 cells were infected with lentiviral particles encoding for shRNA sequence to target ERK5. Levels of indicated proteins were detected by immunoblot analysis. **(E)** The chemical chaperon TUDC ameliorates the UPR and autophagy induced by ERK5i. MiaPaCa-2 cells were treated with 300 μM TUDC for 3 h, previous to incubation with 5 μM JWG-071 for 15 h. Levels of the indicated proteins were evaluated by immunoblotting. Blots representative of two separate experiments. Right histograms show the quantification of CHOP and LC3-II levels, relative to actin, as estimated by densitometry. Values represent mean ± SD of two different determinations. *p < 0.05, ***p < 0.001 from JWG-071 single treatment. **(F)** The chemical chaperone TUDC ameliorates ERK5i-mediated cytotoxicity. Cells were pre-incubated with vehicle or 300 μM TUDC for 3 h before adding 5 μM JWG-071 for further 24 h. Cell viability was determined by MTT assay. Values (mean ± SD) are representative of three different experiments, each performed in tetraplicates. *p < 0.05, ***p < 0.001 from JWG-071 single treatment. **(G)** The ATF4/CHOP axis mediates in ERK5i-induced autophagy. MiaPaCa-2 cells were transfected with scrambled siRNA (siScr) or ATF4-directed siRNA (siATF4), and then treated with vehicle or JWG-071. Protein levels of ATF4, CHOP, LC3 and Actin (loading control) were analyzed by immunoblot. Blots are representative of three independent experiments. Right histograms show the quantification of ATF4 and LC3-II levels, relative to Actin, as estimated by densitometry. Values represent mean ± SD of two different determinations. *p < 0.05 **p < 0.01 ****p < 0.0001 from vehicle-treated cells.

We first performed time-course experiments using JWG-071 in MiaPaCa-2 cells. ERK5 inhibition induced a rapidly (30 min) increased expression of the chaperone BiP, a hallmark of ER stress (Figure 5A). ERK5 inhibition also resulted in a rapid upregulation of the protein levels of the ER stress mediator ATF4 (30 min) and its downstream target CHOP, which preceded to LC3 lipidation (LC3-II) (Figure 5A). We also confirmed activation of ER stress in HeLa and Ishikawa cells by immunoblot analysis (Figure 5B **)**. These results were further confirmed by qRT-PCR analysis. Thus, ERK5 inhibition resulted in a sustained increase on *CHOP* and *ATF4* mRNA levels at 8 and 24 h in MiaPaCa-2 cells and HeLa cells (Figure 5B).

In response to ER stress, IRE1 excises a 26-nucleotide intron of the of transcription factor *XBP1* (X-box binding protein 1 unspliced) RNA, resulting in an unconventional mRNA spliced form (*XBP1s*) that regulates transcription genes involved in the response to ER stress (Yoshida et al., 2001). ERK5 inhibitor induced upregulation of *XBP1s* mRNA levels at 8 h (two-fold) or 24 h (five-fold change) in MiaPaCa-2 cells (Figure 5C), showing that ERK5 inhibition also results in activation of the IRE1/XBP1s branch of the UPR.

Finally, we performed genetic silencing experiments with specific shRNAs. As shown in Figure 5D, ERK5 silencing with specific lentiviral shRNA resulted in increased levels of ATF4 and CHOP proteins. Together, our results demonstrate that impairment of MEK5/ERK5 pathway results in a potent and sustained activation of the UPR in cancer cells.

### ERK5 Inhibition Induces ER Stress-Mediated Cytotoxic Autophagy

To establish the role of the ER stress in the ERK5 inhibition-induced cancer cell death, we next investigated whether ER stress mitigation with chemical chaperones relieved JWG-071-mediated cytotoxicity. For this purpose, we used sodium tauroursodeoxycholicolate acid (TUDC), which attenuates ER stress by promoting protein folding (Cao et al., 2013). TUDC significantly impaired CHOP upregulation and LC3 lipidation in response to ERK5 inhibition (Figure 5E). Furthermore, TUDC mitigated the cytotoxicity induced by JWG-071 in HeLa, Ishikawa and MiaPaCa-2 cancer cells (Figure 5F). Parallel experiments showed that efficient silencing of ATF4 with a specific siRNA resulted in a significant decrease in the ATF4 and LC3-II levels induced by JWG-071 (Figure 5G). These results suggest that ER stress mediates cytotoxic autophagy induced by ERK5 inhibition.

**FIGURE 6:**
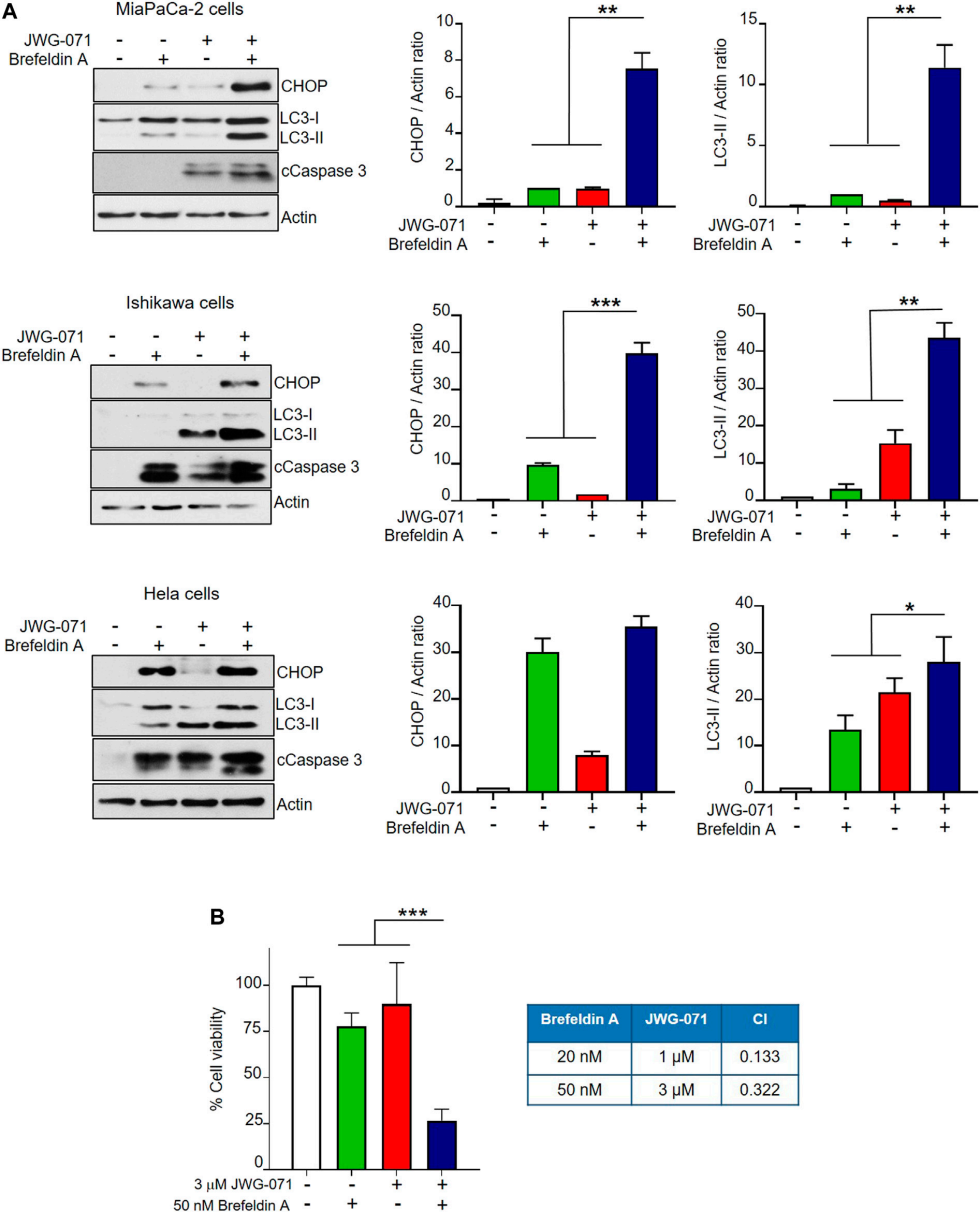
ERK5 inhibition cooperates with the ER stress inducer brefeldin A to activate UPR, autophagy and cytotoxicity. **(A)** Cells were treated for 24 h with 5 μM JWG-071 and/or 400 nM brefeldin A. Levels of the indicated proteins were determined by immunoblot analysis. cCaspase-3, cleaved caspase-3. Right histograms show the quantification of CHOP and LC3-II levels, relative to actin, as estimated by densitometry. Values represent mean ± SD of two different determinations. *p < 0.05 **p < 0.01, ***p < 0.001 from individual treatments with JWG-071 or brefeldin A. **(B)** ERK5i synergizes with brefeldin A to induce cytotoxicity. MTT assay of MiaPaCa-2 cells incubated during 24 h with JWG-071 and/or brefeldin A at the indicated concentrations. Right table show the combination index (CI) analysis for different concentrations of JWG-071 and brefeldin A, obtained using the Compusyn software (CI > 1, antagonism; CI = 1, summary effect; CI < 1, synergism.

Finally, we aimed to determine whether ERK5 inhibition synergizes with canonical ER stressors to induce UPR and autophagy. To this end, we treated MiaPaCa-2, Ishikawa and HeLa cells with JWG-071 and/or brefeldin A. Brefeldin A causes protein accumulation in the ER by disrupting the ER-Golgi transport, thereby leading to ER stress and the correspondent activation of the UPR (Helms and Rothman, 1992). As expected, both JWG-071 and brefeldin A induced UPR (augmented CHOP expression) and autophagy (augmented LC3-II) (Figure 6A). Interestingly, ERK5 inhibition enhanced the effect of brefeldin A on the expression of CHOP and LC3-II, resulting in higher UPR, autophagy and apoptosis (cleaved Caspase 3) (Figure 6A). Consequently, ERK5 inhibition synergized with brefeldin A to promote death in MiaPaCa-2 cells (Figure 6B, see combination index values lower than 1).

## Discussion

Over the last years, the molecular machinery that drives autophagosome biogenesis has been extensively characterized. However, our knowledge on the regulation of autophagy in response to intra- or extracellular stimuli is far from clear, and an integrative vision of autophagy regulation is lacking (Hurtley and Young, 2017). Here, we report for the first time an unexpected role for the MEK5-ERK5 pathway as a novel negative regulator of autophagy in cancer cells. Inhibition of the MEK5/ERK5 pathway with different compounds induces the autophagic flux in a panel of cancer cell lines (Figures 1, 2). Interestingly, the cell lines used in this study present different mutation patterns, including *KRAS* and *TP53* (MiaPaCa-2 pancreatic ductal adenocarcinoma cells), *PTEN* and *TP53* (Ishikawa endometrial adenocarcinoma cells), or *TP53* mutations only (Hela cervical carcinoma cells). Our results suggest that autophagy induction by ERK5 inhibition is independent of Ras/Raf/ERK and PI3K-mTOR oncogenic pathways, two important cellular pathways that have been shown to regulate autophagy in cancer cells (Zada et al., 2021). Numerous studies have shown that ERK5 inhibitors have antitumor activity by activating apoptosis in cancer cells and in tumor xenografts (reviewed in Gomez et al. (2016), Hoang et al. (2017), Stecca and Rovida (2019)). In this work, we show that autophagy mediates apoptosis induced by ERK5 inhibition, and therefore, the antitumor activity of the ERK5 inhibitors (Figure 3).

Our results suggest that ERK5 inhibition stimulates the autophagic flux independently of the primary autophagy regulators AMPK and mTORC1 (Figure 4). Consistently, levels and phosphorylation status of the autophagy master regulator ULK/ATG1 at Ser555 and Ser757 were not affected by ERK5 inhibition (Figure 4). However, we cannot rule out the effect of ERK5 inhibition on other kinases that, phosphorylating different residues on ULK, might activate autophagy. For instance, it has been recently reported that PKCα modulates ULK activity by phosphorylating Ser423 (Wang et al., 2018). Also, ERK5 could participate on autophagy by direct phosphorylation of components of the autophagic machinery, as previously reported for other MAPK family members. For instance, ERK8/MAPK15 promotes autophagy by direct phosphorylation of ULK1 (Colecchia et al., 2018), and JNK1 participates in the activation of the PI3KC3 complex (a key regulator of autophagosome formation) by phosphorylating the Beclin 1 inhibitor Bcl-2 (Wei et al., 2008). Conversely, it will be important to investigate whether autophagy-related ATG proteins cross-regulate ERK5 phosphorylation/activity, as reported for ERK2 (Martinez-Lopez et al., 2013) and p38 (Quiang et al., 2013). In this regard, it will be important to investigate whether ERK5 phosphorylates key proteins of the autophagic machinery.

**FIGURE 7:**
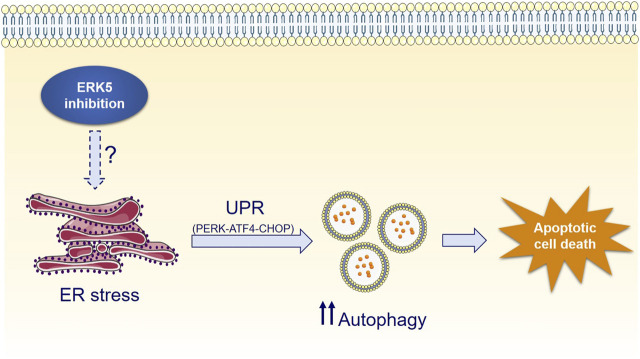
Mechanism of ERK5i-induced cytotoxic autophagy.

Interestingly, ERK5 inhibition or silencing induced ER stress and sustained expression of the UPR markers CHOP, ATF4 and of the spliced form of XBP-1, whereas mitigation of UPR by chemical chaperones impaired JWG-071-mediated cytotoxicity (Figures 5A–D). Furthermore, genetic suppression of the ATF4/CHOP axis partially abrogates ERK5 inhibition-induced autophagy (Figure 5E), while the canonical ER stress inductor brefeldin A cooperates with JWG-071 to exacerbate UPR, autophagy and cytotoxicity (Figure 6). These results suggest that activation of the UPR pathway in response to ER stress mediates (at least partially) the autophagy and cytotoxicity induced by ERK5 inhibitors (see Figure 7 for a model of mechanism of action of ERK5i).

In line with our results, the ERK5 orthologue in *S. cerevisiae* Slt2p/Mpk1p is activated during ER stress, and this activation mediates cell survival during the UPR induced in response to ER stress (Bonilla and Cunningham, 2003; Chen et al., 2005). Here, we show by first time that ERK5 inhibition results in activation of ER stress and the UPR in human cancer cells (JWG-071 induced upregulation of the chaperone BiP, Figure 4A). Regarding the UPR, it has been shown that ERK5 inhibition activates the UPR pathway in pancreatic beta-cells, and that CHOP deficiency ameliorates ERK5 inhibition-mediated exacerbation of streptozotocin toxicity (Nam et al., 2017). On the contrary, active ERK5 mediates the suppression of ER stress and UPR necessary for the anti-apoptotic effect of neuroprotectors dexmedetomidine and Netrin-1 after cerebral ischemia injury (Yin et al., 2021). Finally, a recent analysis of global transcriptome (RNA-Seq) showed that CRISPR/Cas9 knockout ERK5 human osteosarcoma U2OS cells express elevated levels of *CHOP*, *TRIB3* an *XBP-1* mRNAs, compared to wild type U2OS cells (Craig et al., 2020). Together, these observations support our evidences showing that ERK5 pathway is involved in UPR activation.

Our work further shows that activation of the UPR ATF4/CHOP axis mediates cellular autophagy activation in response to ERK5 inhibition (Figures 5F,G). Yet, the precise molecular mechanism by which ERK5 modulates UPR and autophagy remains unanswered. Recently, the MEK5/ERK5 pathway was reported to be a positive regulator of mitophagy. Genetic o pharmacological inhibition of the MEK5/ERK5 pathway increases the mitochondrial content by impairing the lysosomal degradation of mitochondria. This could result in increased defective mitochondria and generation of reactive oxygen species (ROS), which could give rise to ER stress and UPR activation (Cao and Kaufman, 2014). In this regard, Liu et al. reported that loss of ERK5 in murine cardiomyocytes (cardiac-specific deletion of ERK5) leads to mitochondrial aberrations and increased production of ROS caused by oxidative damage (Liu et al., 2017). Hence, it will be interesting to explore whether ERK5 inhibitors activate ER stress and the UPR via generating ROS from altered mitochondrial function.

Autophagy is activated in response to stress or nutrient deprivation, including ER stress, to mitigate damage and provide nutrients for cellular survival. In response to ER stress, activation of cellular autophagy eliminates abnormal protein aggregates, thus contributing to cellular homeostasis recovery (Hetz, 2012; Rashid et al., 2015). However, the outcome of autophagy is highly dependent on the intensity and duration of the stimuli, and persistent activation can lead to cytotoxic autophagy (Marino et al., 2014). Cancer cells are particularly dependent on accurate sensing of stress cues and their energetic status, and they largely rely on precise responses to these inputs. Specifically, cancer cells have developed high dependency on the UPR and autophagy to overcome limiting tumor conditions and to maintain their high metabolic rate within the hostile tumor microenvironment (Chen and Cubillos-Ruiz, 2021). To this end, cancer cells express high levels of some of the UPR genes (like BiP and ATF6), which allow them a better tolerance against environment stress (Shuda et al., 2003). Here, we show that pharmacological and genetic suppression of the MEK5/ERK5 pathway markedly increased the UPR and the autophagic flux in cancer cells, indicating that ERK5 activity contributes to fine-tuning of UPR and autophagy in this context (Figures 1, 2). Conversely, overexpression of either MEK5 or ERK5 did not influence basal levels of UPR or autophagy (data not shown), suggesting that basal ERK5 activity is enough to restrain autophagy to a moderate rate. It is therefore intriguing to hypothesize that ERK5 levels or catalytic activity could be intrinsically subjected to stress-dependent modulation as a mechanism to regulate autophagy levels in cancer.

While UPR and autophagy typically display cytoprotective functions, they actually display a dual role in cell fate decisions. Specifically, the balance between the pro-survival and pro-death faces of the ER stress and the UPR is determined by their duration and intensity. When persistent UPR fails to relieve ER stress, sustained increased levels of the CHOP transcription factor may invoke the pro-apoptotic arm of the UPR, resulting in the so call ER-mediated apoptosis (Lu et al., 2014; Hetz and Papa, 2018). This seems to be the case for ERK5 inhibition of cancer cells, since it resulted in CHOP overexpression and XBP-1 splicing after 24 h, and in increased apoptosis (Figure 5). Similarly, a sustained and exacerbated autophagic flux elicits varied mechanisms to activate cell death pathways in case the cellular damage remains, leading to the so called autophagy-mediated cell death (Yonekawa and Thorburn, 2013). Accordingly, pharmacologic manipulation of UPR and autophagy has recently arisen as a potential tool for improving anticancer therapies (Bialik et al., 2018). For example, the lipid-derived small molecule ABTL0812—which is currently in clinical trial for the treatment of advanced endometrial (NCT02201823) and ductal pancreatic cancer (NCT04431258)—induces cytotoxic autophagy (Erazo et al., 2016) by provoking a sustained activation of the UPR (Munoz-Guardiola et al., 2020). Similar behaviour has been reported for the cannabinoid Δ^9^-tetrahydrocannabinol (THC), which has antitumor activity in glioblastoma by triggering autophagy-mediated apoptosis by a mechanism that involves ER stress and the UPR (Salazar et al., 2009). This could also be the case for ERK5 inhibitors, which induced a robust and sustained UPR activation (al least of 48 h) that lead to activation of caspase-3 in cancer cell lines. Because ERK5 inhibition did not induce cytotoxicity in autophagy-deficient transformed MEF cells (Figures 3F,G), our results support the notion that ERK5 modulation induces autophagy-dependent apoptotic cell death in cancer cells (Figure 7).

The unique duality of ERK5 inhibition to impair cancer cell proliferation and to induce cytotoxic autophagy underlines the anticancer potential of ERK5 inhibitors, and could have important clinical implications. Given the fact that ERK5 inhibition sensitizes cancer cells and tumors to different chemotherapies (Pereira et al., 2016; Pereira and Rodrigues, 2020), future work will be necessary to determine the relevance of the UPR and autophagy in the combined used of chemotherapy and ERK5 inhibitors to tackle cancer.

## Data Availability

The original contributions presented in the study are included in the article/Supplementary Material, further inquiries can be directed to the corresponding author.

## References

[B1] Bialik S. Dasari S. K. Kimchi A. (2018). Autophagy-Dependent Cell Death - Where, How and Why a Cell Eats Itself to Death. J. Cell Sci. 131. 10.1242/jcs.215152 30237248

[B2] Bonilla M. Cunningham K. W. (2003). Mitogen-Activated Protein Kinase Stimulation of Ca(2+) Signaling is Required for Survival of Endoplasmic Reticulum Stress in Yeast. Mol. Biol. Cell 14, 4296–4305. 10.1091/mbc.e03-02-0113 14517337 PMC207020

[B3] Brodsky J. L. Skach W. R. (2011). Protein Folding and Quality Control in the Endoplasmic Reticulum: Recent Lessons from Yeast and Mammalian Cell Systems. Curr. Opin. Cell Biol. 23, 464–475. 10.1016/j.ceb.2011.05.004 21664808 PMC3154734

[B4] Calfon M. Zeng H. Urano F. Till J. H. Hubbard S. R. Harding H. P. (2002). IRE1 Couples Endoplasmic Reticulum Load to Secretory Capacity by Processing the XBP-1 mRNA. Nature 415, 92–96. 10.1038/415092a 11780124

[B5] Cao S. S. Kaufman R. J. (2014). Endoplasmic Reticulum Stress and Oxidative Stress in Cell Fate Decision and Human Disease. Antioxid. Redox Signal. 21, 396–413. 10.1089/ars.2014.5851 24702237 PMC4076992

[B6] Cao S. S. Zimmermann E. M. Chuang B. M. Song B. Nwokoye A. Wilkinson J. E. (2013). The Unfolded Protein Response and Chemical Chaperones Reduce Protein Misfolding and Colitis in Mice. Gastroenterology 144, 989–1000. 10.1053/j.gastro.2013.01.023 23336977 PMC3751190

[B7] Chen X. Cubillos-Ruiz J. R. (2021). Endoplasmic Reticulum Stress Signals in the Tumour and its Microenvironment. Nat. Rev. Cancer 21, 71–88. 10.1038/s41568-020-00312-2 33214692 PMC7927882

[B8] Chen Y. Feldman D. E. Deng C. Brown J. A. De Giacomo A. F. Gaw A. F. (2005). Identification of Mitogen-Activated Protein Kinase Signaling Pathways that Confer Resistance to Endoplasmic Reticulum Stress in *Saccharomyces cerevisiae* . Mol. Cancer Res. 3, 669–677. 10.1158/1541-7786.mcr-05-0181 16380504

[B9] Chou T. (2010). Drug Combination Studies and Their Synergy Quantification Using the Chou-Talalay Method. Cancer Res. 70, 440–460. 10.1158/0008-5472.can-09-1947 20068163

[B10] Cirone M. Gilardini Montani M. S. Granato M. Garufi A. Faggioni A. D'Orazi G. (2019). Autophagy Manipulation as a Strategy for Efficient Anticancer Therapies: Possible Consequences. J. Exp. Clin. Cancer Res. 38, 262. 10.1186/s13046-019-1275-z 31200739 PMC6570888

[B11] Colecchia D. Dapporto F. Tronnolone S. Salvini L. Chiariello M. (2018). MAPK15 Is Part of the ULK Complex and Controls its Activity to Regulate Early Phases of the Autophagic Process. J. Biol. Chem. 293, 15962–15976. 10.1074/jbc.ra118.002527 30131341 PMC6187625

[B12] Craig J. E. Miller J. N. Rayavarapu R. R. Hong Z. Bulut G. B. Zhuang W. (2020). MEKK3-MEK5-ERK5 Signaling Promotes Mitochondrial Degradation. Cell Death Discov 6, 107. 10.1038/s41420-020-00342-7 33101709 PMC7576125

[B13] Erazo T. Espinosa-Gil S. Dieguez-Martinez N. Gomez N. Lizcano J. M. (2020). SUMOylation is Required for ERK5 Nuclear Translocation and ERK5-Mediated Cancer Cell Proliferation. Int. J. Mol. Sci. 21 (6), 2203. 10.3390/ijms21062203 PMC7139592 32209980

[B14] Erazo T. Lorente M. Lopez-Plana A. Munoz-Guardiola P. Fernandez-Nogueira P. Garcia-Martinez J. A. (2016). The New Antitumor Drug ABTL0812 Inhibits the Akt/mTORC1 Axis by Upregulating Tribbles-3 Pseudokinase. Clin. Cancer Res. 22, 2508–2519. 10.1158/1078-0432.ccr-15-1808 26671995

[B15] Erazo T. Moreno A. Ruiz-Babot G. Rodriguez-Asiain A. Morrice N. A. Espadamala J. (2013). Canonical and Kinase Activity-independent Mechanisms for Extracellular Signal-Regulated Kinase 5 (ERK5) Nuclear Translocation Require Dissociation of Hsp90 from the ERK5-Cdc37 Complex. Mol. Cell Biol. 33, 1671–1686. 10.1128/mcb.01246-12 23428871 PMC3624243

[B16] Glick D. Barth S. Macleod K. F. (2010). Autophagy: Cellular and Molecular Mechanisms. J. Pathol. 221, 3–12. 10.1002/path.2697 20225336 PMC2990190

[B17] Gomez N. Erazo T. Lizcano J. M. (2016). ERK5 and Cell Proliferation: Nuclear Localization is What Matters. Front. Cell Dev. Biol. 4, 105. 10.3389/fcell.2016.00105 27713878 PMC5031611

[B18] He C. Klionsky D. J. (2009). Regulation Mechanisms and Signaling Pathways of Autophagy. Annu. Rev. Genet. 43, 67–93. 10.1146/annurev-genet-102808-114910 19653858 PMC2831538

[B19] Helms J. B. Rothman J. E. (1992). Inhibition by Brefeldin A of a Golgi Membrane Enzyme that Catalyses Exchange of Guanine Nucleotide Bound to ARF. Nature 360, 352–354. 10.1038/360352a0 1448152

[B20] Hernandez-Tiedra S. Fabrias G. Davila D. Salanueva I. J. Casas J. Montes L. R. (2016). Dihydroceramide Accumulation Mediates Cytotoxic Autophagy of Cancer Cells via Autolysosome Destabilization. Autophagy 12, 2213–2229. 10.1080/15548627.2016.1213927 27635674 PMC5103338

[B21] Hetz C. Papa F. R. (2018). The Unfolded Protein Response and Cell Fate Control. Mol. Cell 69, 169–181. 10.1016/j.molcel.2017.06.017 29107536

[B22] Hetz C. (2012). The Unfolded Protein Response: Controlling Cell Fate Decisions under ER Stress and beyond. Nat. Rev. Mol. Cell Biol. 13, 89–102. 10.1038/nrm3270 22251901

[B23] Hoang V. T. Yan T. J. Cavanaugh J. E. Flaherty P. T. Beckman B. S. Burow M. E. (2017). Oncogenic Signaling of MEK5-ERK5. Cancer Lett. 392, 51–59. 10.1016/j.canlet.2017.01.034 28153789 PMC5901897

[B71] Hurtley J. H. Young L. N. (2017). Mechanisms of Autophagy Initiation. Annu. Rev. Biochem. 86, 225–244. 10.1146/annurev-biochem-061516-044820 28301741 PMC5604869

[B24] Jain B. P. (2017). An Overview of Unfolded Protein Response Signaling and its Role in Cancer. Cancer Biother. Radiopharm. 32, 275–281. 10.1089/cbr.2017.2309 29053418

[B25] Kabeya Y. Mizushima N. Yamamoto A. Oshitani-Okamoto S. Ohsumi Y. Yoshimori T. (2004). LC3, Gabarap and Gate16 Localize to Autophagosomal Membrane Depending on Form-II Formation. J. Cell Sci. 117, 2805–2812. 10.1242/jcs.01131 15169837

[B26] Kasler H. G. Victoria J. Duramad O. Winoto A. (2000). ERK5 Is a Novel Type of Mitogen-Activated Protein Kinase Containing a Transcriptional Activation Domain. Mol. Cell Biol. 20, 8382–8389. 10.1128/mcb.20.22.8382-8389.2000 11046135 PMC102145

[B27] Kato Y. Kravchenko V. V. Tapping R. I. Han J. Ulevitch R. J. Lee J. D. (1997). Bmk1/ERK5 Regulates Serum-Induced Early Gene Expression through Transcription Factor MEF2C. EMBO J. 16, 7054–7066. 10.1093/emboj/16.23.7054 9384584 PMC1170308

[B28] Kato Y. Tapping R. I. Huang S. Watson M. H. Ulevitch R. J. Lee J. D. (1998). Bmk1/ERK5 is Required for Cell Proliferation Induced by Epidermal Growth Factor. Nature 395, 713–716. 10.1038/27234 9790194

[B29] Kim J. Kundu M. Viollet B. Guan K. L. (2011). AMPK and mTOR Regulate Autophagy through Direct Phosphorylation of Ulk1. Nat. Cell Biol. 13, 132–141. 10.1038/ncb2152 21258367 PMC3987946

[B30] Klionsky D. J. Abdel-Aziz A. K. Abdelfatah S. Abdellatif M. Abdoli A. Abel S. (2021). Guidelines for the Use and Interpretation of Assays Fro Monitoring Autophagy (4th Edition). Autophagy 17, 1–382. 10.1080/15548627.2020.1797280 33634751 PMC7996087

[B31] Klionsky D. J. Ohsumi Y. (1999). Vacuolar Import of Proteins and Organelles from the Cytoplasm. Annu. Rev. Cell Dev. Biol. 15, 1–32. 10.1146/annurev.cellbio.15.1.1 10611955

[B32] Levine B. Kroemer G. (2019). Biological Functions of Autophagy Genes: A Disease Perspective. Cell 176, 11–42. 10.1016/j.cell.2018.09.048 30633901 PMC6347410

[B33] Lin E. C. K. Amantea C. A. Nomanbhoy T. K. Weissig H. Ishiyama J. Hu Y. (2016). ERK5 Kinase Activity Is Dispensable for Cellular Immune Response and Proliferation. Proc. Natl. Acad. USA 113, 11865–11870. 10.1073/pnas.1609019113 PMC5081620 27679845

[B34] Liu C. Y. Schroder M. Kaufman R. J. (2000). Ligand-independent Dimerization Activates the Stress Response Kinases IRE1 and PERK in the Lumen of the Endoplasmic Reticulum. J. Biol. Chem. 275, 24881–24885. 10.1074/jbc.m004454200 10835430

[B35] Liu W. Ruiz-Velasco A. Wang S. Khan S. Zi M. Jungmann A. (2017). Metabolic Stress-Induced Cardiomyopathy is Caused by Mitochondrial Dysfunction Due to Attenuated Erk5 Signaling. Nat. Commun. 8, 494. 10.1038/s41467-017-00664-8 28887535 PMC5591279

[B36] Lu M. Lawrence D. A. Marsters S. Acosta-Alvear D. Kimmig P. Mendez A. S. (2014). Opposing Unfolded-Protein-Response Signals Converge on Death Receptor 5 to Control Apoptosis. Science 345, 98–101. 10.1126/science.1254312 24994655 PMC4284148

[B37] Marino G. Niso-Santano M. Baehrecke E. H. Kroemer G. (2014). Self-consumption: the Interplay of Autophagy and Apoptosis. Nat. Rev. Mol. Cell Biol. 15, 81–94. 10.1038/nrm3735 24401948 PMC3970201

[B38] Martinez-Lopez N. Athonvarangkul D. Mishall P. Sahu S. Singh R. (2013). Autophagy Proteins Regulate ERK Phosphorylation. Nat. Commun. 4, 2799. 10.1038/ncomms3799 24240988 PMC3868163

[B39] McCullough K. D. Martindale J. L. Klotz L. O. Aw T. Y. Holbrook N. J. (2001). Gadd153 Sensitizes Cells to Endoplasmic Reticulum Stress by Down-Regulating Bcl2 and Perturbing the Cellular Redox State. Mol. Cell Biol. 21, 1249–1259. 10.1128/mcb.21.4.1249-1259.2001 11158311 PMC99578

[B40] Munoz-Guardiola P. Casas J. Megias-Roda E. Sole S. Perez-Montoyo H. Yeste-Velasco M. (2020). The Anti-Cancer Drug ABTL0812 Induces ER Stress-Mediated Cytotoxic Autophagy by Increasing Dihydroceramide Levels in Cancer Cells. Autophagy, 1–18. 10.1080/15548627.2020.1761651 PMC8204958 32397857

[B41] Nam D. H. Han J. H. Lim J. H. Park K. M. Woo C. H. (2017). CHOP Deficiency Ameliorates ERK5 Inhibition-Mediated Exacerbation of Streptozotocin-Induced Hyperglycemia and Pancreatic Beta-Cell Apoptosis. Mol. Cell 40, 457–465. 10.14348/molcells.2017.2296 PMC5547215 28681594

[B42] Pereira D. M. Rodrigues C. M. P. (2020). Targeted Avenues for Cancer Treatment: The MEK5-ERK5 Signaling Pathway. Trends Mol. Med. 26, 394–407. 10.1016/j.molmed.2020.01.006 32277933

[B43] Pereira D. M. Simoes A. E. Gomes S. E. Castro R. E. Carvalho T. Rodrigues C. M. (2016). MEK5/ERK5 Signaling Inhibition Increases colon Cancer Cell Sensitivity to 5-fluorouracil through a P53-dependent Mechanism. Oncotarget 7, 34322–34340. 10.18632/oncotarget.9107 27144434 PMC5085159

[B44] Puissant A. Robert G. Fenouille N. Luciano F. Cassuto J. P. Raynaud S. (2010). Resveratrol Promotes Autophagic Cell Death in Chronic Myelogenous Leukemia Cells via JNK-Mediated p62/SQSTM1 Expression and AMPK Activation. Cancer Res. 70, 1042–1052. 10.1158/0008-5472.can-09-3537 20103647

[B45] Puthalakath H. O'Reilly L. A. Gunn P. Lee L. Kelly P. N. Huntington N. D. (2007). ER Stress Triggers Apoptosis by Activating BH3-Only Protein Bim. Cell 129, 1337–1349. 10.1016/j.cell.2007.04.027 17604722

[B46] Quiang L. Wu C. Ming M. Viollet B. He Y. Y. (2013). Autophgay Controls P38 Activation to Promote Cell Survival under Genotoxic Stress. J. Bio. Chem. 288, 1603–1611. 10.1074/jbc.M112.415224 23212914 PMC3548470

[B47] Rashid H. O. Yadav R. K. Kim H. R. Chae H. J. (2015). ER Stress: Autophagy Induction, Inhibition and Selection. Autophagy 11, 1956–1977. 10.1080/15548627.2015.1091141 26389781 PMC4824587

[B48] Razumovskaya E. Sun J. Ronnstrand L. (2011). Inhibition of MEK5 by BIX02188 Induces Apoptosis in Cells Expressing the Oncogenic Mutant FLT3-ITD. Biochem. Biophys. Res. Commun. 412, 307–312. 10.1016/j.bbrc.2011.07.089 21820407

[B49] Romanov J. Walczak M. Ibiricu I. Schüchner S. Ogris E. Kraft C. (2012). Mechanism and Functions of Membrane Binding by the Atg5-Atg12/Atg16 Complex during Autophagosome Formation. EMBO J 31, 4304–4317. 10.1038/emboj.2012.278 23064152 PMC3501226

[B50] Salazar M. Carracedo A. Salanueva I. J. Hernandez-Tiedra S. Lorente M. Egia A. (2009). Cannabinoid Action Induces Autophagy-Mediated Cell Death through Stimulation of ER Stress in Human Glioma Cells. J. Clin. Invest. 119, 1359–1372. 10.1172/jci37948 19425170 PMC2673842

[B51] Schroder M. Kaufman R. J. (2005). The Mammalian Unfolded Protein Response. Annu. Rev. Biochem. 74, 739–789. 10.1146/annurev.biochem.73.011303.074134 15952902

[B52] Shore G. C. Papa F. R. Oakes S. A. (2011). Signaling Cell Death from the Endoplasmic Reticulum Stress Response. Curr. Opin. Cell Biol. 23, 143–149. 10.1016/j.ceb.2010.11.003 21146390 PMC3078187

[B53] Shuda M. Kondoh N. Imazeki N. Tanaka K. Okada T. Mori K. (2003). Activation of the ATF6, XBP1 and Grp78 Genes in Human Hepatocellular Carcinoma: a Possible Involvement of the ER Stress Pathway in Hepatocarcinogenesis. J. Hepatol. 38, 605–614. 10.1016/s0168-8278(03)00029-1 12713871

[B54] Singh S. S. Vats S. Chia A. Y. Tan T. Z. Deng S. Ong M. S. (2018). Dual Role of Autophagy in Hallmarks of Cancer. Oncogene 37, 1142–1158. 10.1038/s41388-017-0046-6 29255248

[B55] Stecca B. Rovida E. (2019). Impact of ERK5 on the Hallmarks of Cancer. Int. J. Mol. Sci. 20 (6), 1426. 10.3390/ijms20061426 PMC6471124 30901834

[B56] Virgin H. W. Levine B. (2009). Autophagy Genes in Immunity. Nat. Immunol. 10, 461–470. 10.1038/ni.1726 19381141 PMC2715365

[B57] Walter P. Ron D. (2011). The Unfolded Protein Response: from Stress Pathway to Homeostatic Regulation. Science 334, 1081–1086. 10.1126/science.1209038 22116877

[B58] Wang J. Erazo T. Ferguson F. M. Buckley D. L. Gomez N. Munoz-Guardiola P. (2018). Structural and Atropisomeric Factors Governing the Selectivity of Pyrimido-Benzodiazipinones as Inhibitors of Kinases and Bromodomains. ACS Chem. Biol. 13, 2438–2448. 10.1021/acschembio.7b00638 30102854 PMC6333101

[B59] Wei Y. Pattingre S. Sinha S. Bassik M. Levine B. (2008). JNK1-mediated Phosphorylation of Bcl-2 Regulates Starvation-Induced Autophagy. Mol. Cell 30, 678–688. 10.1016/j.molcel.2008.06.001 18570871 PMC2478643

[B60] Yamamoto K. Sato T. Matsui T. Sato M. Okada T. Yoshida H. (2007). Transcriptional Induction of Mammalian ER Quality Control Proteins Is Mediated by Single or Combined Action of ATF6alpha and XBP1. Dev. Cell 13, 365–376. 10.1016/j.devcel.2007.07.018 17765680

[B61] Yang Q. Deng X. Lu B. Cameron M. Fearns C. Patricelli M. P. (2010). Pharmacological Inhibition of BMK1 Suppresses Tumor Growth through Promyelocytic Leukemia Protein. Cancer Cell 18, 258–267. 10.1016/j.ccr.2010.08.008 20832753 PMC2939729

[B62] Yao Z. Yoon S. Kalie E. Raviv Z. Seger R. (2010). Calcium Regulation of EGF-Induced ERK5 Activation: Role of Lad1-MEKK2 Interaction. PLoS One 5, e12627. 10.1371/journal.pone.0012627 20830310 PMC2935384

[B63] Yin J. W. Li J. Ren Y. M. Li Y. Wang R. X. Wang S. (2021). Dexmedetomidine and Netrin-1 Combination Therapy Inhibits Endoplasmic Reticulum Stress by Regulating the ERK5/MEF2A Pathway to Attenuate Cerebral Ischemia Injury. Front. Neurosci. 15, 641345. 10.3389/fnins.2021.641345 33584197 PMC7876398

[B64] Yin Z. Pascual C. Klionsky D. J. (2016). Autophagy: Machinery and Regulation. Microb. Cell 3, 588–596. 10.15698/mic2016.12.546 28357331 PMC5348978

[B65] Yonekawa T. Thorburn A. (2013). Autophagy and Cell Death. Essays Biochem. 55, 105–117. 10.1042/bse0550105 24070475 PMC3894632

[B66] Yoshida H. Matsui T. Yamamoto A. Okada T. Mori K. (2001). XBP1 mRNA Is Induced by ATF6 and Spliced by IRE1 in Response to ER Stress to Produce a Highly Active Transcription Factor. Cell 107, 881–891. 10.1016/s0092-8674(01)00611-0 11779464

[B67] Zachari M. Ganley I. G. (2017). The Mammalian ULK1 Complex and Autophagy Initiation. Essays Biochem. 61, 585–596. 10.1042/ebc20170021 29233870 PMC5869855

[B68] Zada S. Hwang J. S. Ahmed M. Lai T. H. Pham T. M. Elashkar O. (2021). Cross Talk between Autophagy and Oncogenic Signaling Pathways and Implications for Cancer Therapy. Biochim. Biophys. Acta Rev. Cancer 1876, 188565. 10.1016/j.bbcan.2021.188565 33992723

[B69] Zhang J. Chiu J. Zhang H. Qi T. Tang Q. Ma K. (2013). Autophagic Cell Death Induced by Resveratrol Depends on the Ca(2+)/AMPK/mTOR Pathway in A549 Cells. Biochem. Pharmacol. 86, 317–328. 10.1016/j.bcp.2013.05.003 23680031

[B70] Zhou G. Bao Z. Q. Dixon J. E. (1995). Components of a New Human Protein Kinase Signal Transduction Pathway. J. Biol. Chem. 270, 12665–12669. 10.1074/jbc.270.21.12665 7759517

