# Supplementary material for: ERK5 Inhibition Induces Autophagy-Mediated Cancer Cell Death by Activating ER Stress
Source: Front Cell Dev Biol. 2021 Nov 4;9:742049. doi: 10.3389/fcell.2021.742049 (PMC8600073; doi:10.3389/fcell.2021.742049)
Supplement: Supplementary file 1 [file DataSheet1.pdf]

Figure 1A

Hela cells

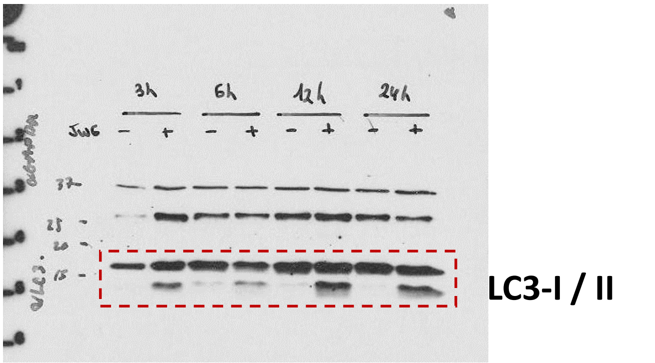

Hela cells

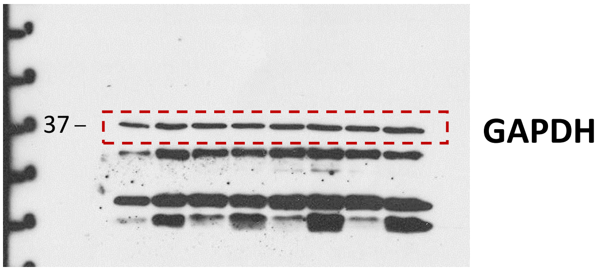

Ishikawa cells

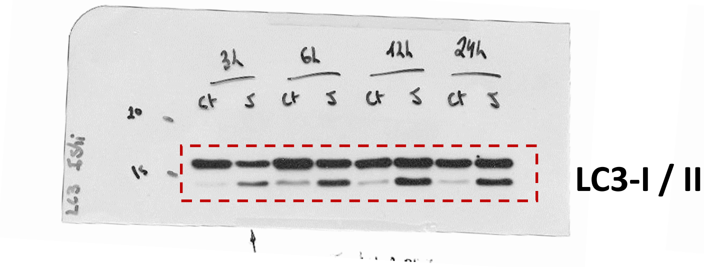

Ishikawa cells

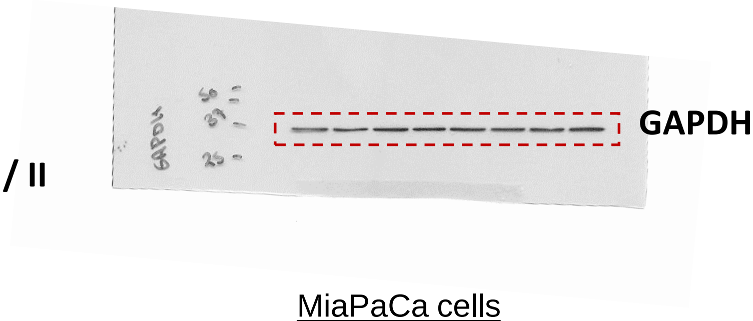

MiaPaCa cells

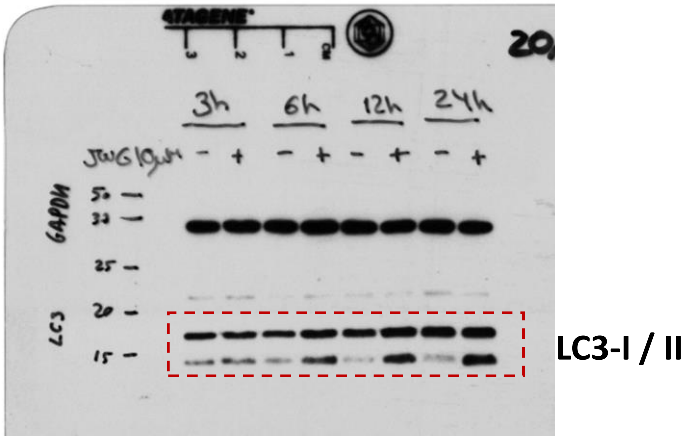

MiaPaCa cells

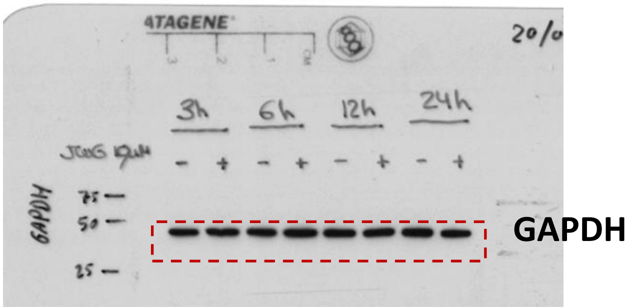

Figure 1B

Hela cells

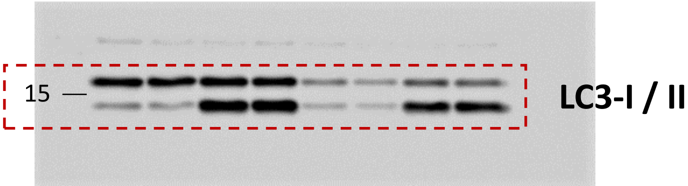

Hela cells

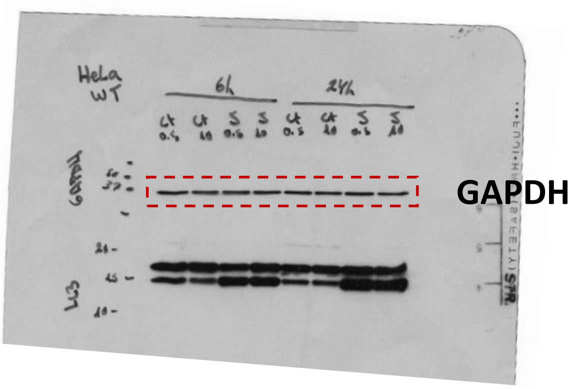

Figure 1C

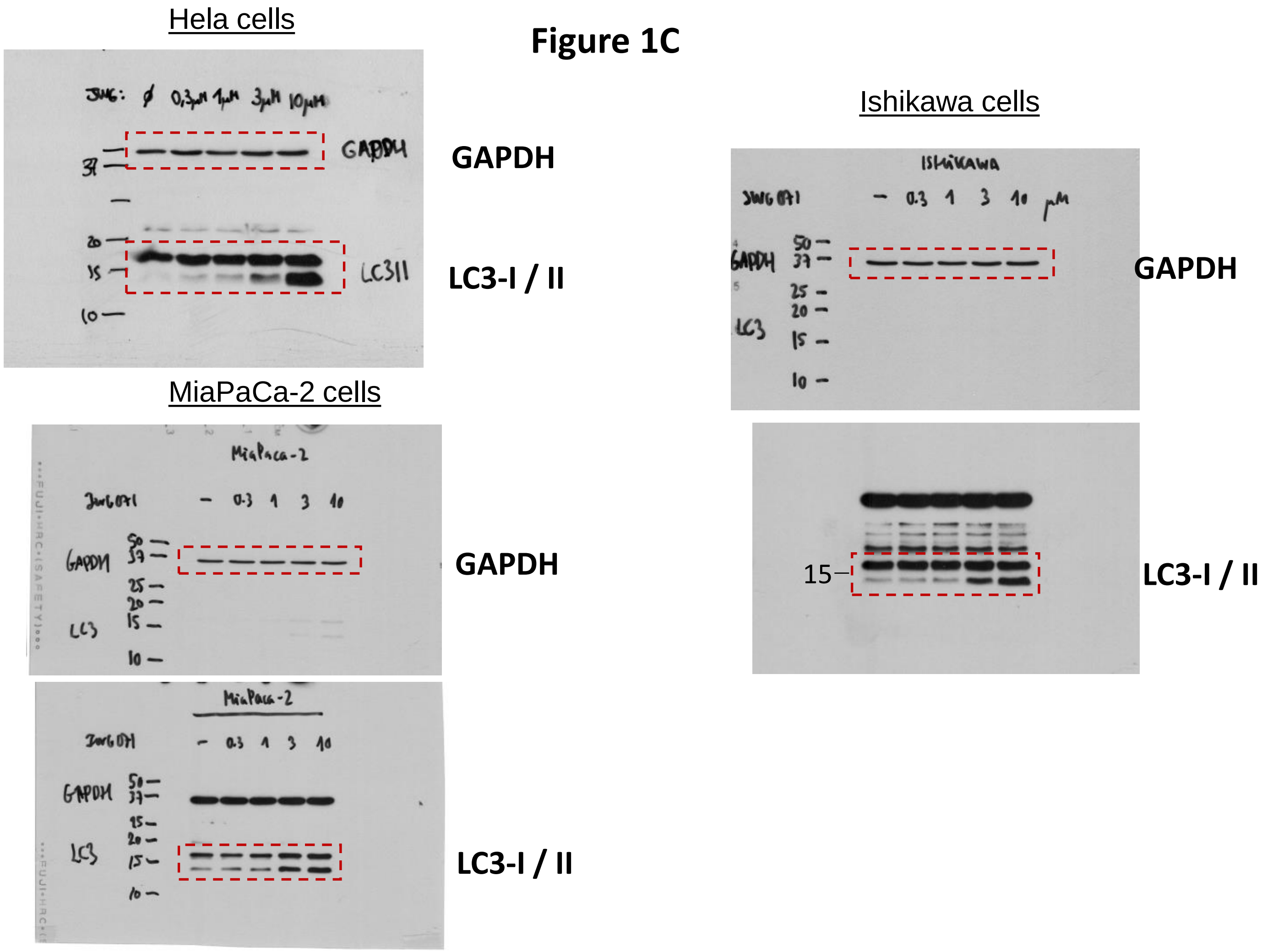

Figure 1D

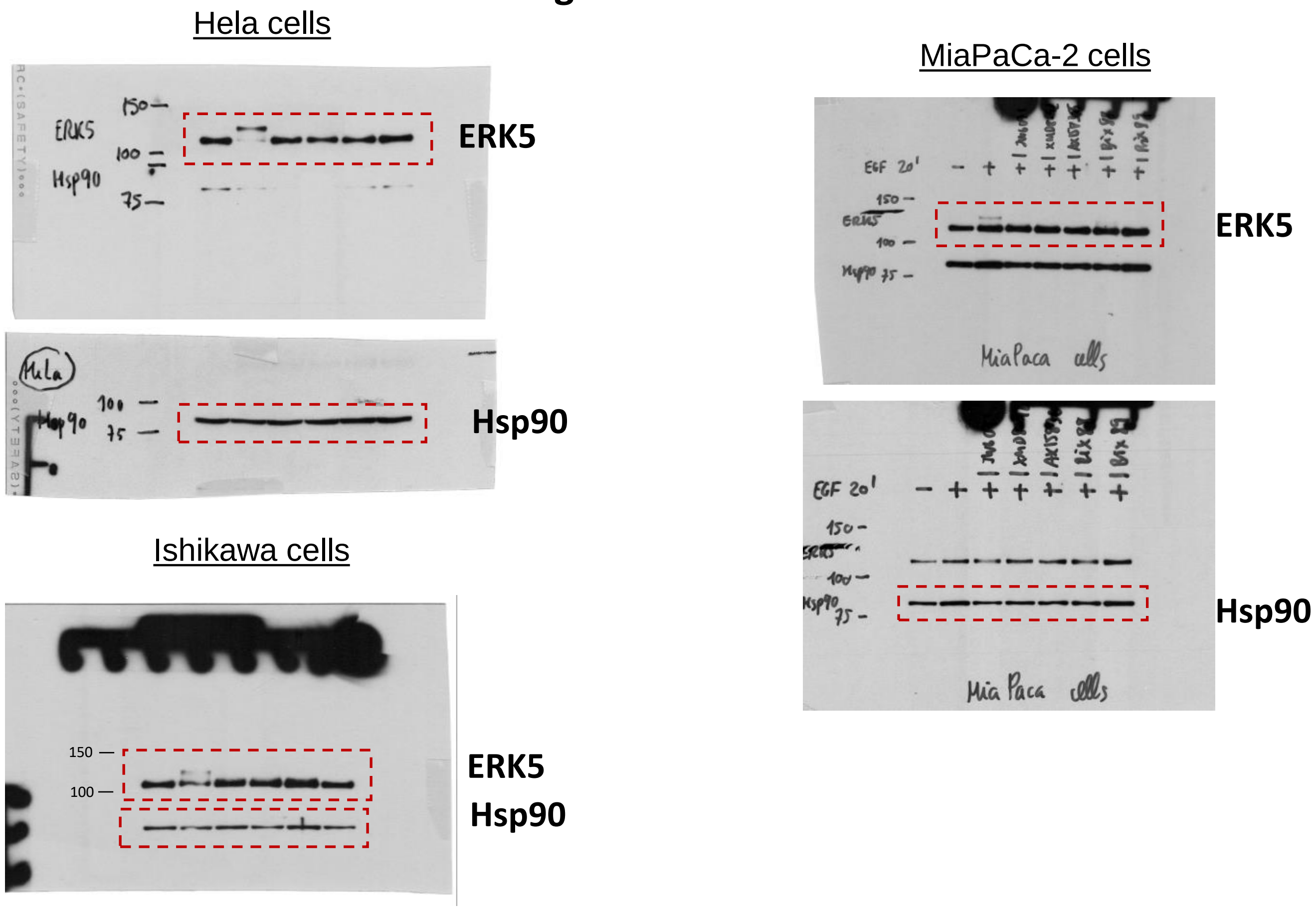

Figure 1E

Ishikawa cells

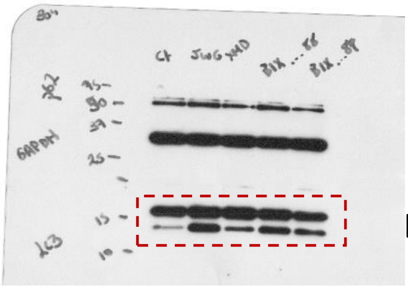

LC3-I / II

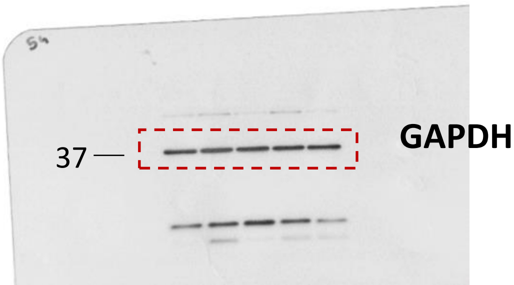

GAPDH

Figure 1F

MiaPaCa-2 cells

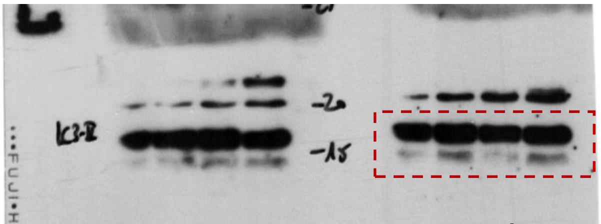

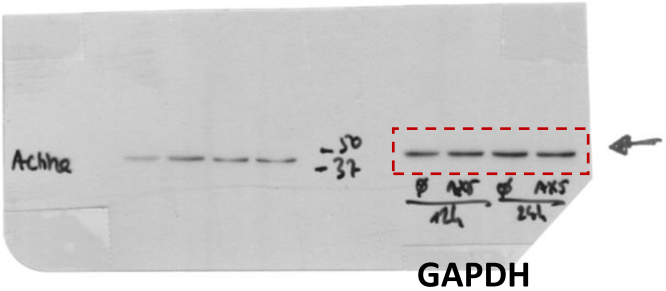

GAPDH

Figure 1G

MiaPaCa-2 cells

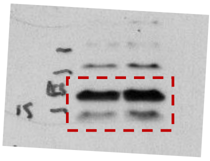

LC3-I / II

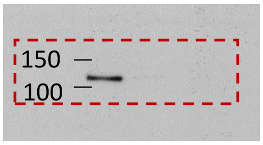

ERK5

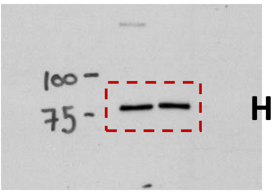

Hsp90

Figure 2B

Ishikawa cells

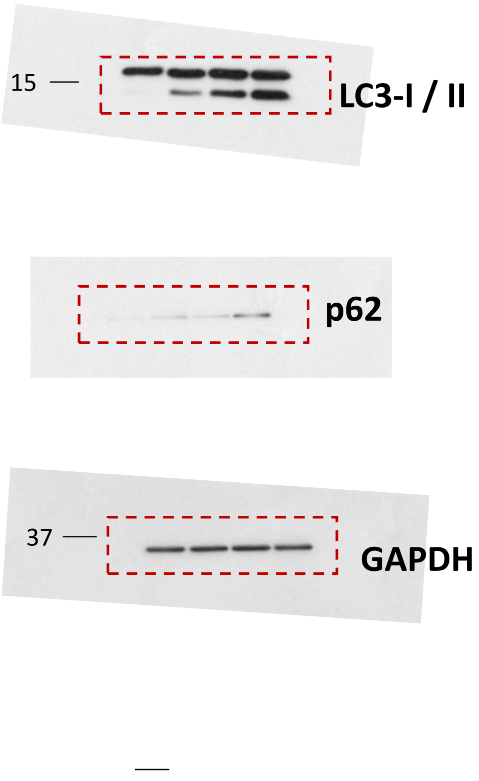

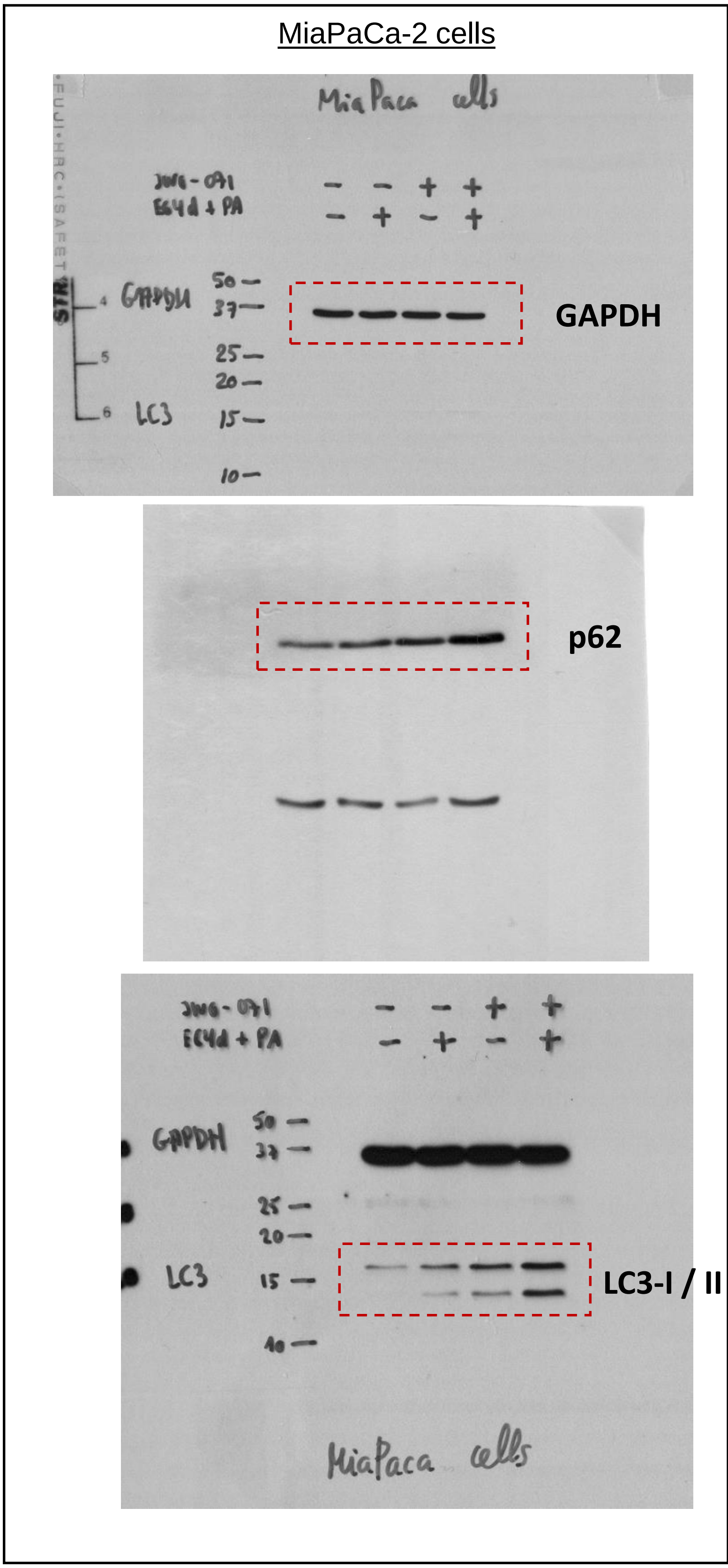

Figure 3C

Hela cells

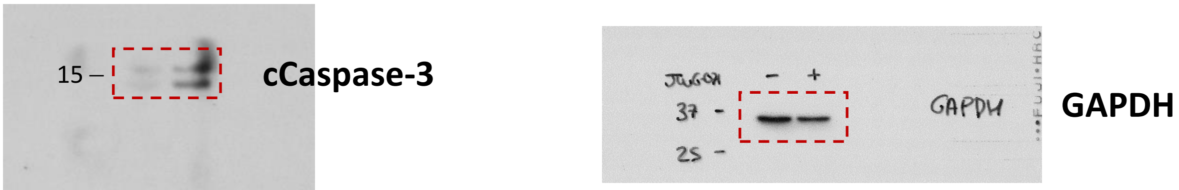

Ishikawa cells

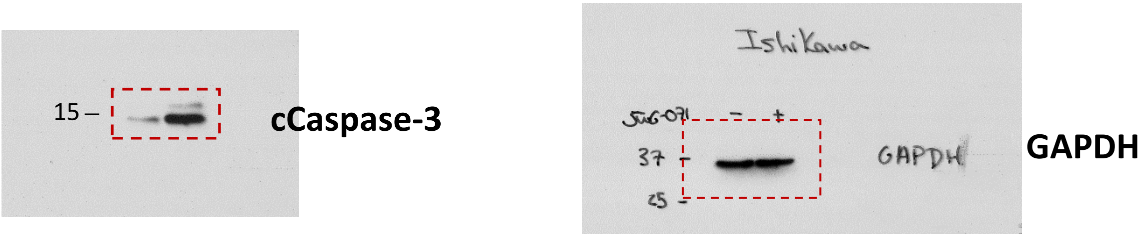

MiaPaCa-2 cells

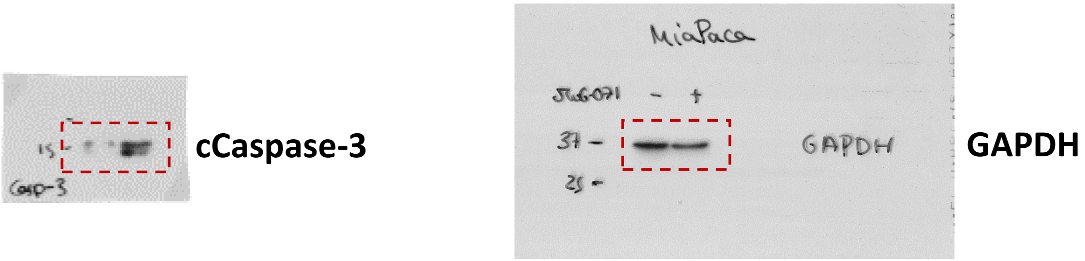

Figure 3D

Hela cells

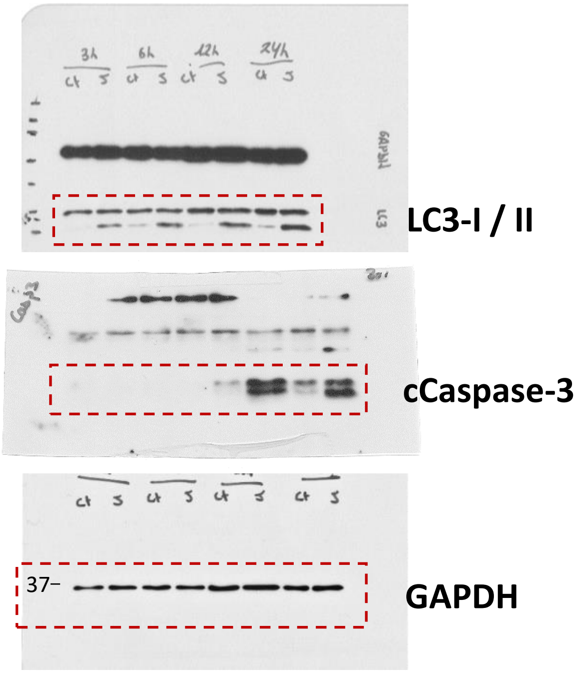

Ishikawa cells

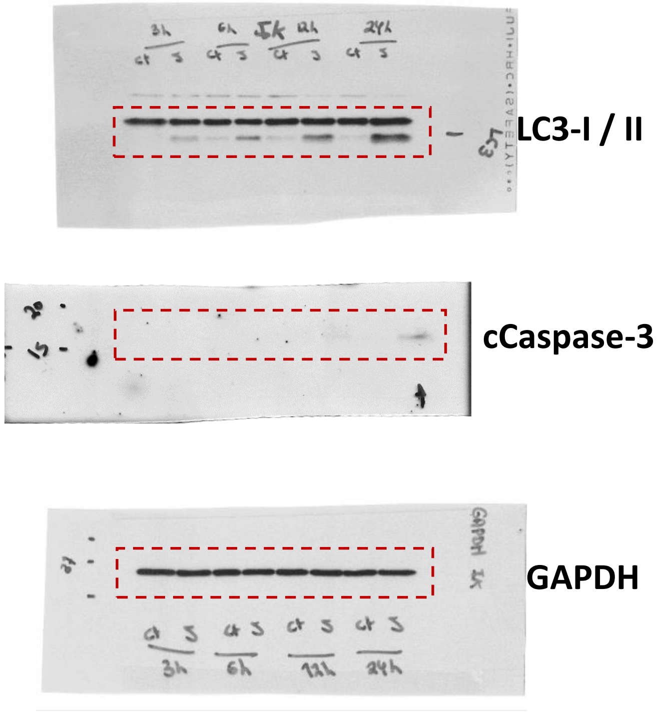

Figure 3E

MEF ATG5 KO + MEF ATG5 WT

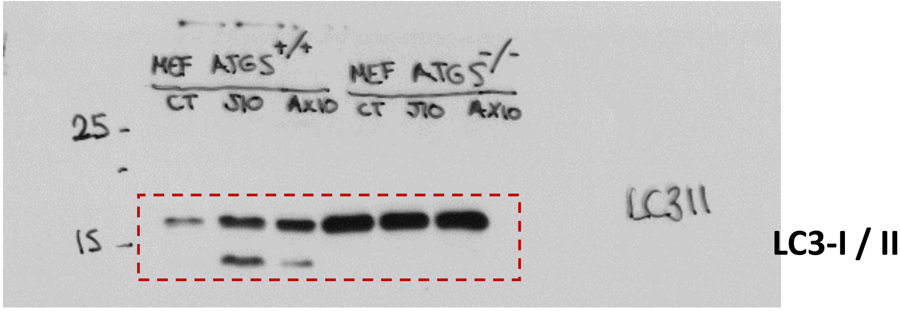

LC3-I / II

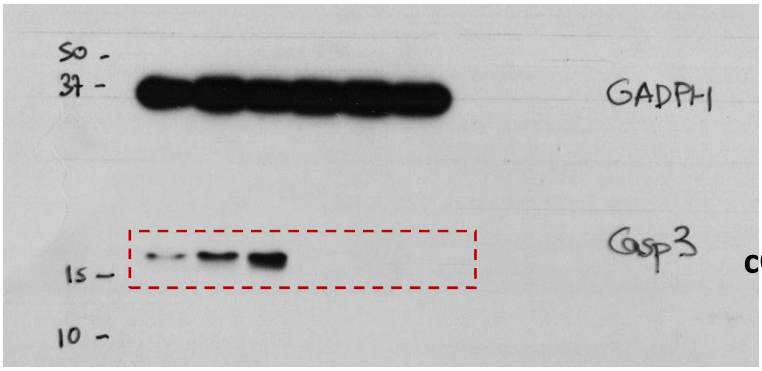

cCaspase-3

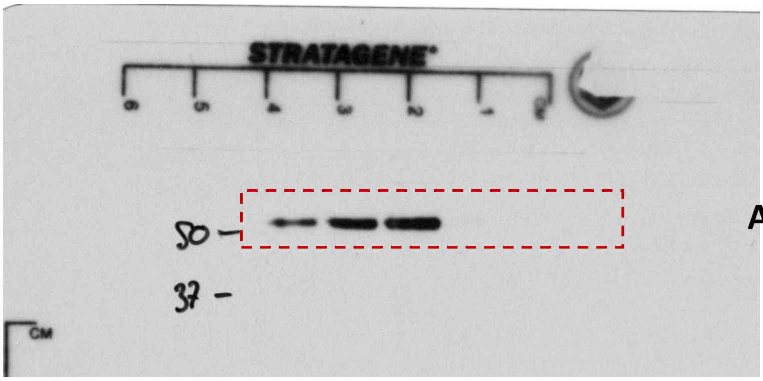

ATG5/ATG12

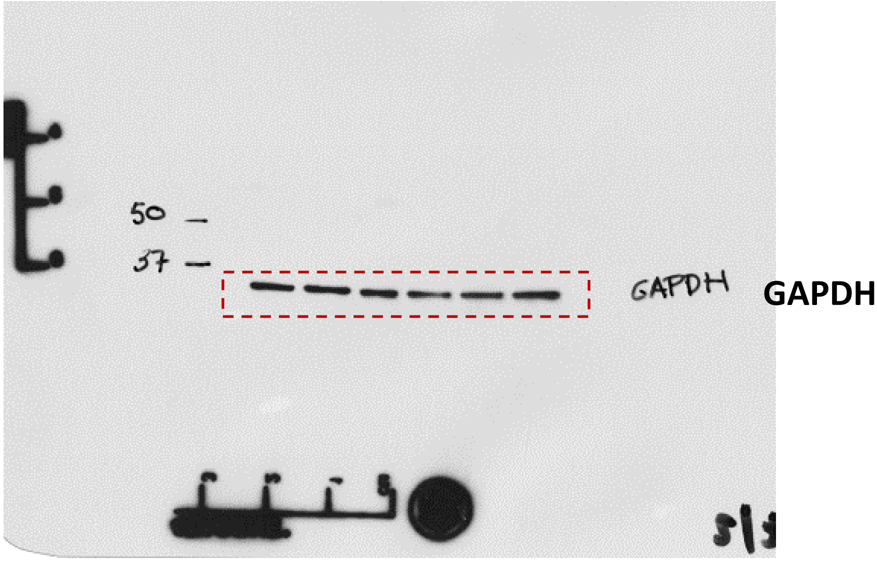

GAPDH

Figure 4A

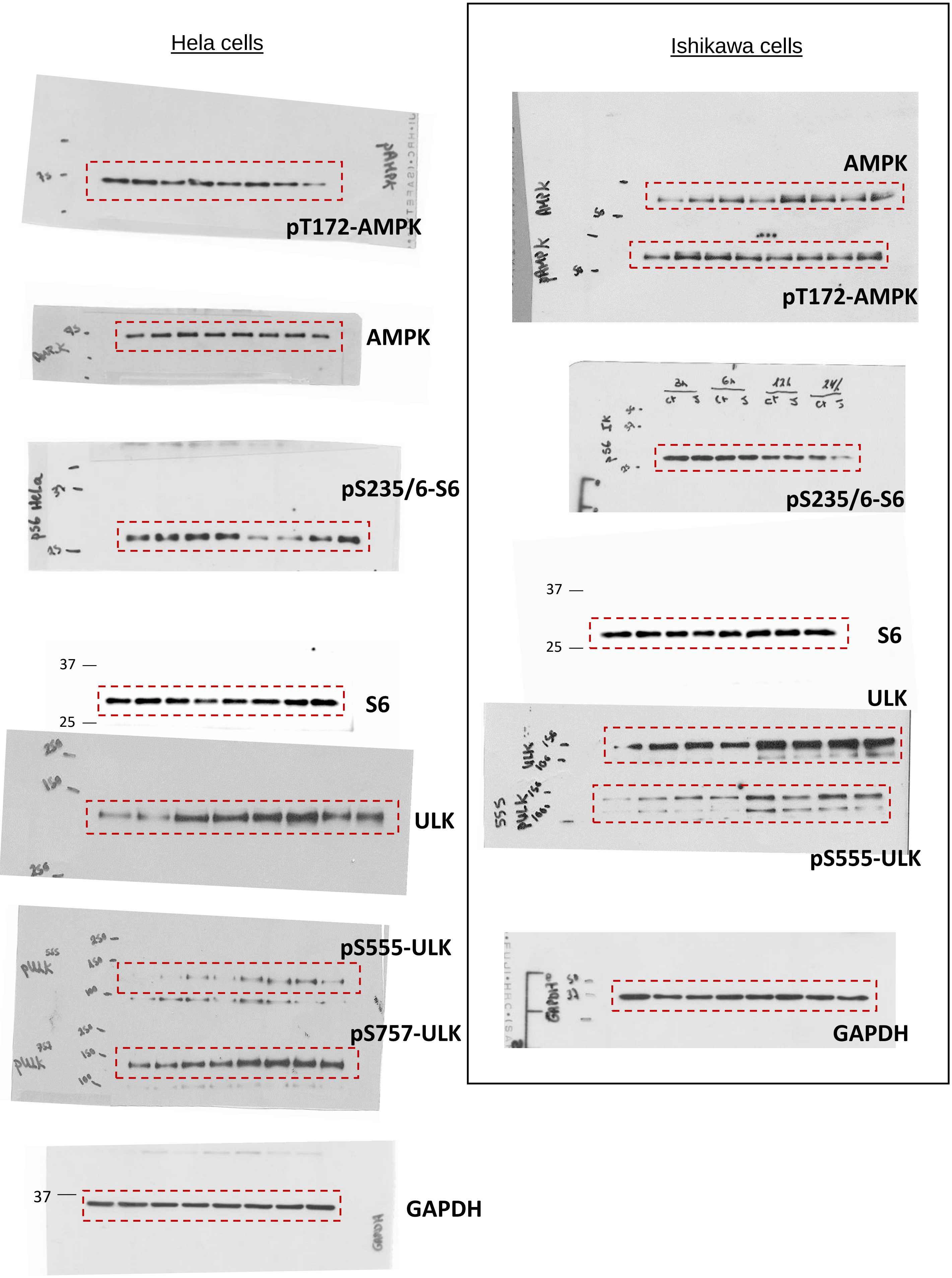
Hela cells
pT172-AMPK
AMPK
pS235/6-S6
37
S6
25
ULK
pS555-ULK
pS757-ULK
37
GAPDH
Ishikawa cells
AMPK
pT172-AMPK
pS235/6-S6
37
S6
25
ULK
pS555-ULK
GAPDH

Figure 5A

<u>MiaPaCa-2 cells</u>

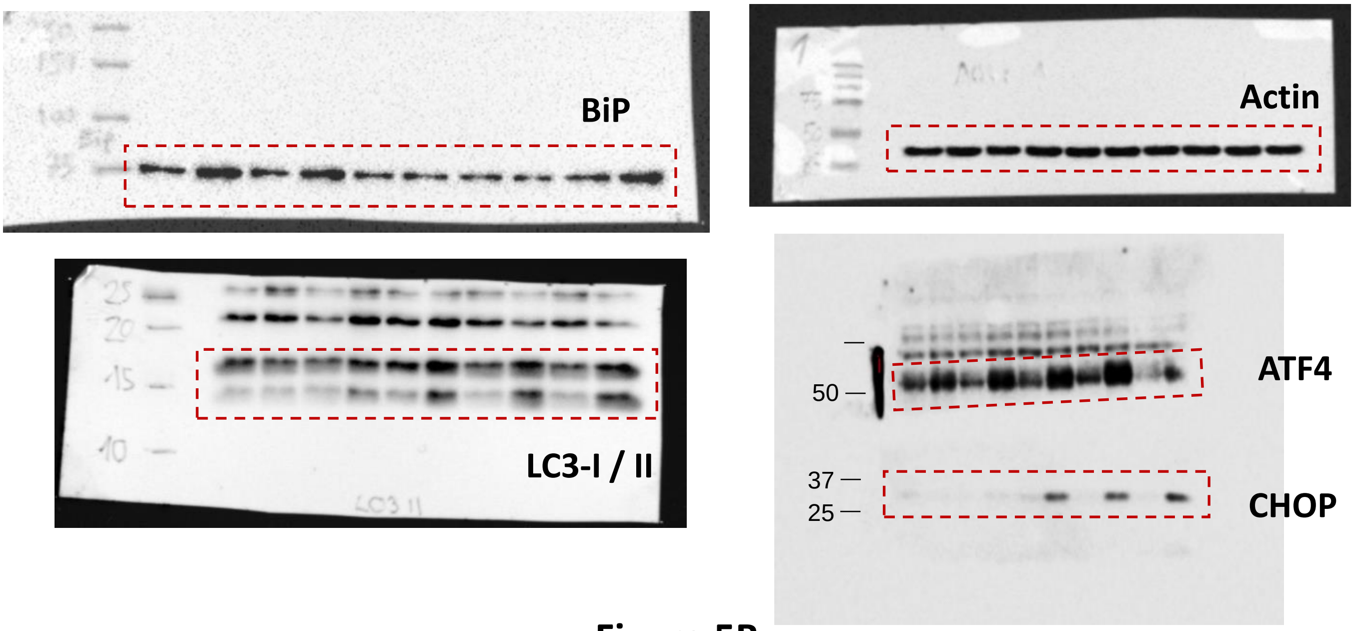

Figure 5B

<u>Ishikawa cells</u>

<u>Hela cells</u>

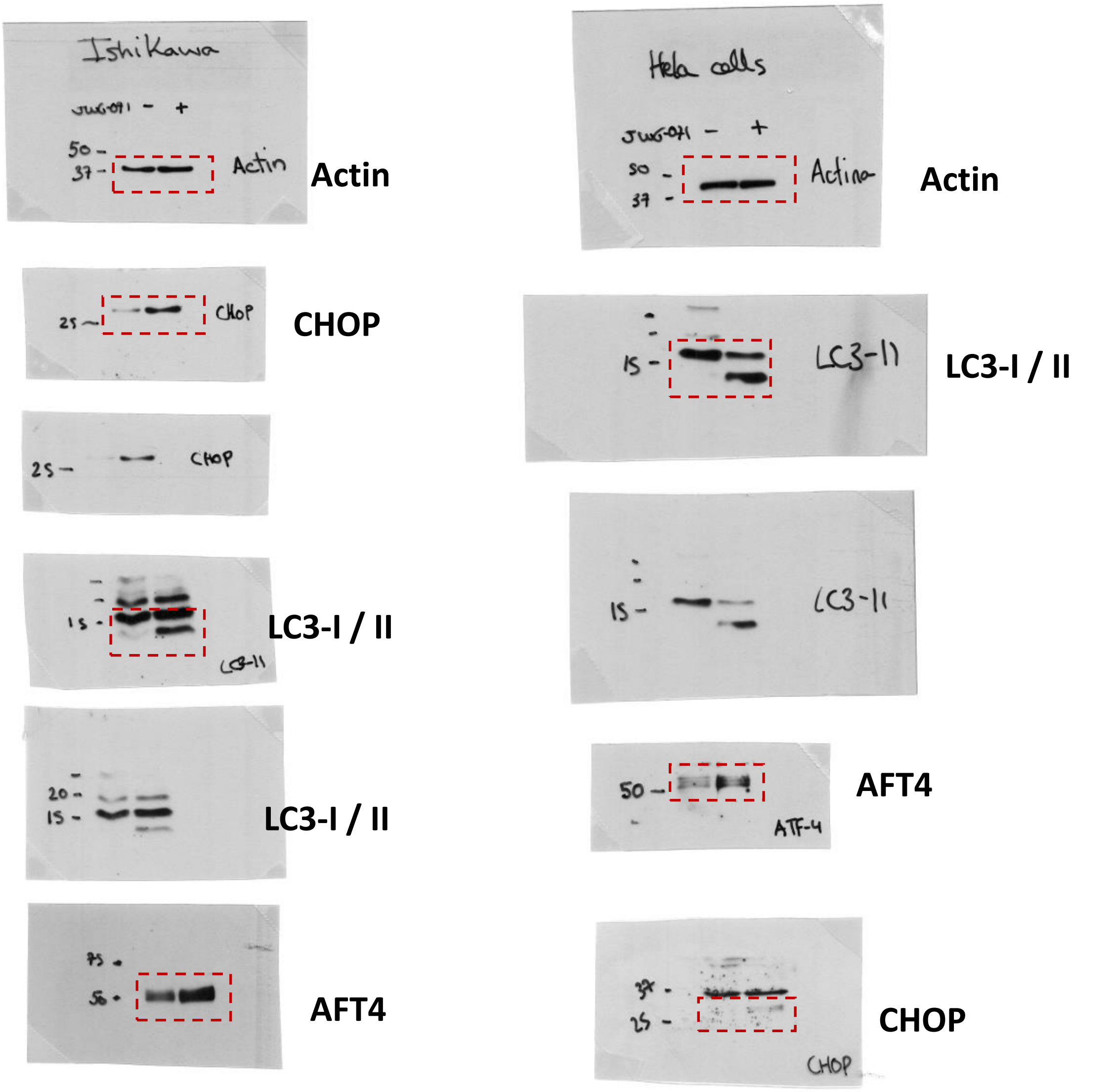

Figure 5D

MiaPaCa-2 cells

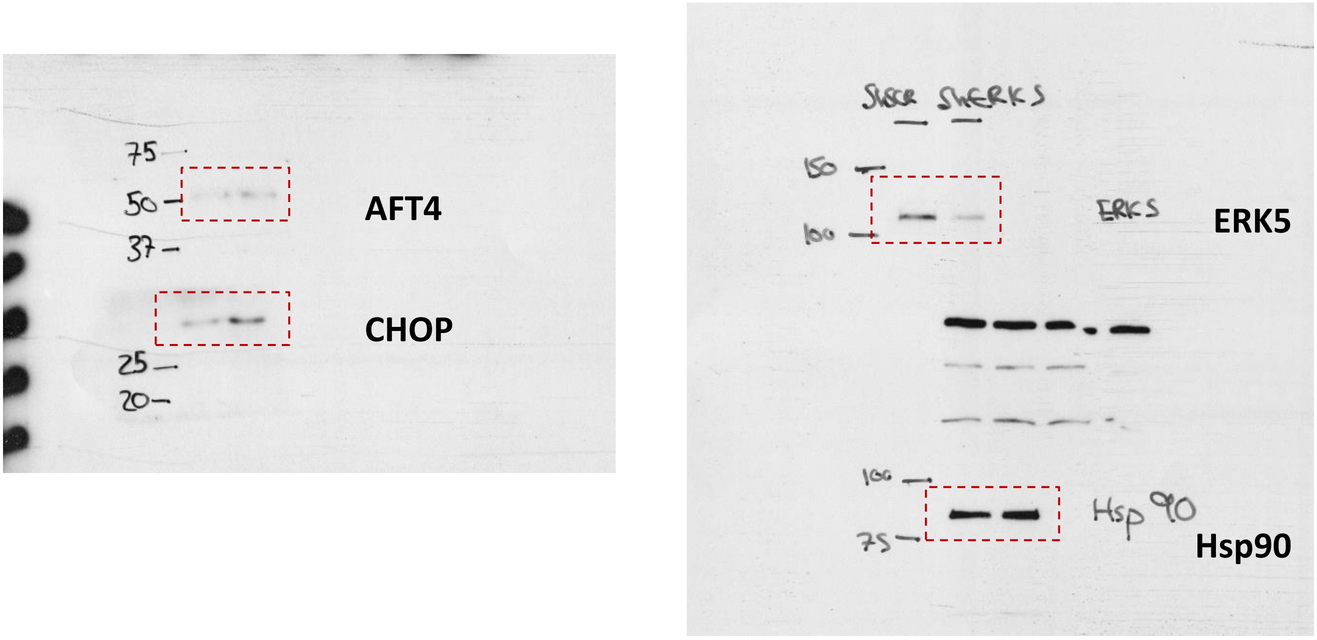

Figure 5E

MiaPaCa-2 cells

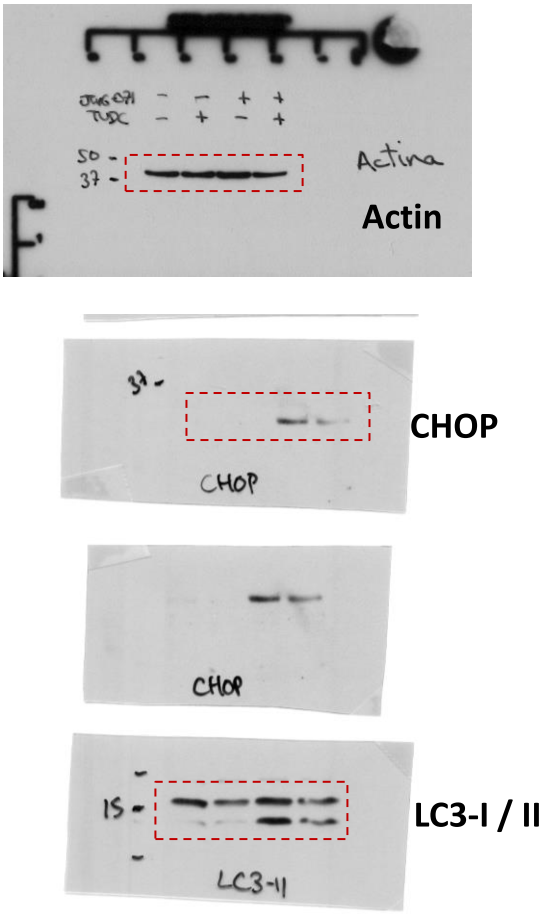

Figure 5G

MiaPaCa-2 cells

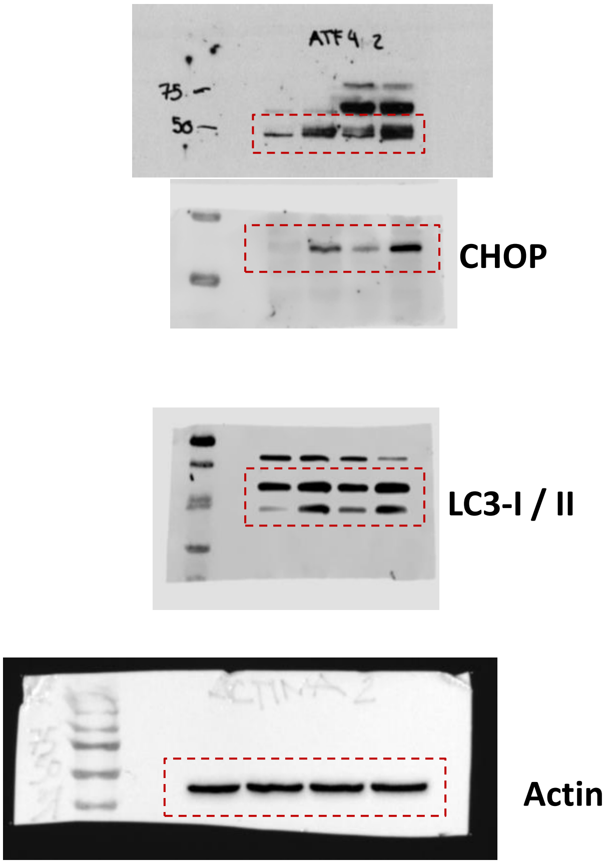

Figure 6

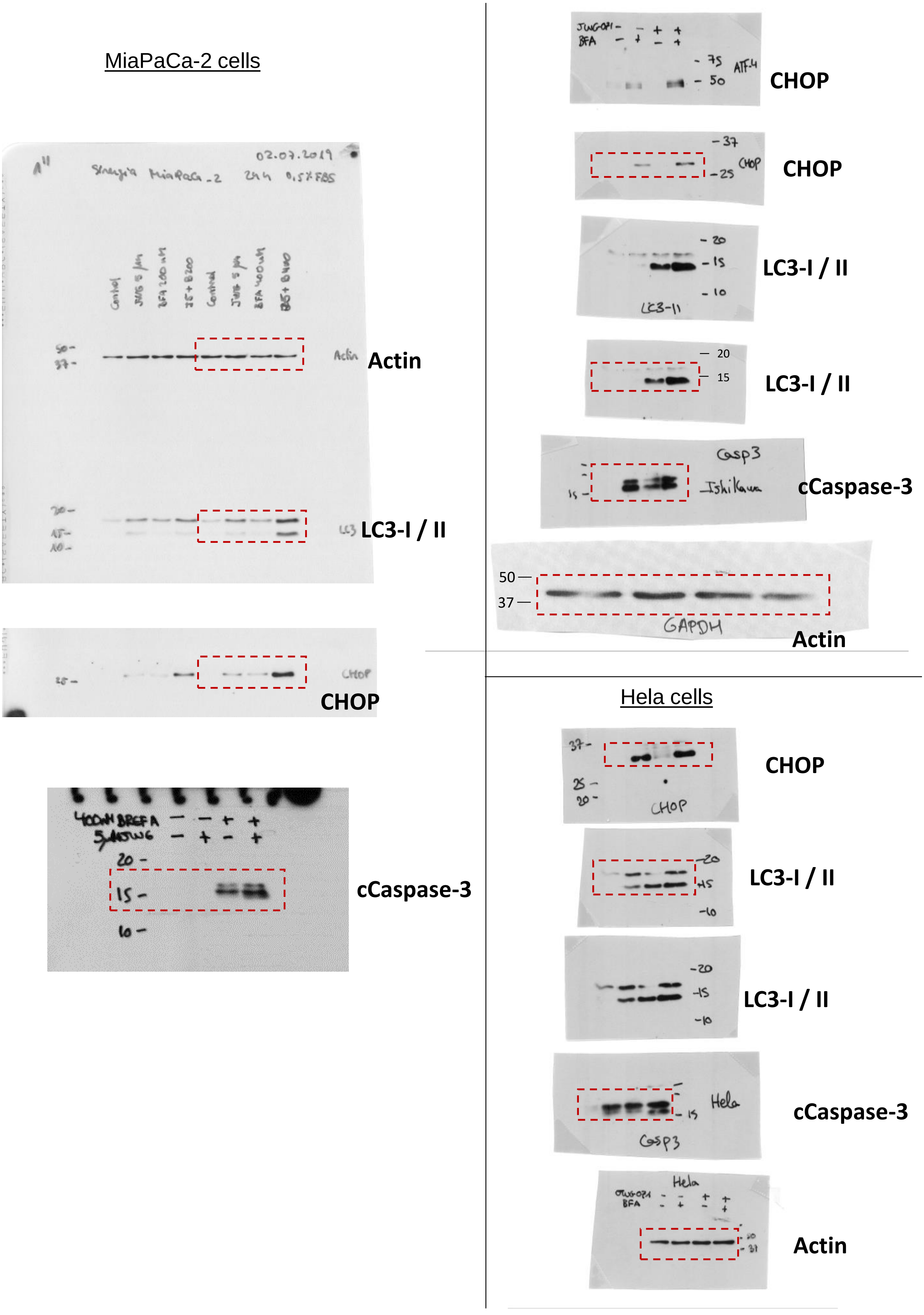

Ishikawa cells
MiaPaCa-2 cells
Hela cells
CHOP
CHOP
LC3-I / II
LC3-I / II
cCaspase-3
Actin
Actin
LC3-I / II
CHOP
cCaspase-3
CHOP
LC3-I / II
LC3-I / II
cCaspase-3
Actin
20
15
50
37
GAPDH
